# The healthiness and sustainability of national and global food based dietary guidelines: modelling study

**DOI:** 10.1136/bmj.m2322

**Published:** 2020-07-15

**Authors:** Marco Springmann, Luke Spajic, Michael A Clark, Joseph Poore, Anna Herforth, Patrick Webb, Mike Rayner, Peter Scarborough

**Affiliations:** 1 Oxford Martin Programme on the Future of Food and Nuffield Department of Population Health, University of Oxford, Oxford OX3 7LF, UK; 2 School of Medicine, University of Adelaide, Adelaide, SA, Australia; 3 Department of Zoology and School of Geography and the Environment, University of Oxford, Oxford, UK; 4 Department of Global Health and Population, Harvard TH Chan School of Public Health, Boston, MA, USA; 5 Friedman School of Nutrition Science and Policy, Tufts University, Boston, MA, USA; 6 Oxford Martin Programme on the Future of Food, NIHR Biomedical Research Centre at Oxford, and Nuffield Department of Population Health, University of Oxford, Oxford, UK

## Abstract

**Objective:**

To analyse the health and environmental implications of adopting national food based dietary guidelines (FBDGs) at a national level and compared with global health and environmental targets.

**Design:**

Modelling study.

**Setting:**

85 countries.

**Participants:**

Population of 85 countries.

**Main outcome measures:**

A graded coding method was developed and used to extract quantitative recommendations from 85 FBDGs. The health and environmental impacts of these guidelines were assessed by using a comparative risk assessment of deaths from chronic diseases and a set of country specific environmental footprints for greenhouse gas emissions, freshwater use, cropland use, and fertiliser application. For comparison, the impacts of adopting the global dietary recommendations of the World Health Organization and the EAT-Lancet Commission on Healthy Diets from Sustainable Food Systems were also analysed. Each guideline’s health and sustainability implications were assessed by modelling its adoption at both the national level and globally, and comparing the impacts to global health and environmental targets, including the Action Agenda on Non-Communicable Diseases, the Paris Climate Agreement, the Aichi biodiversity targets related to land use, and the sustainable development goals and planetary boundaries related to freshwater use and fertiliser application.

**Results:**

Adoption of national FBDGs was associated with reductions in premature mortality of 15% on average (95% uncertainty interval 13% to 16%) and mixed changes in environmental resource demand, including a reduction in greenhouse gas emissions of 13% on average (regional range −34% to 35%). When universally adopted globally, most of the national guidelines (83, 98%) were not compatible with at least one of the global health and environmental targets. About a third of the FBDGs (29, 34%) were incompatible with the agenda on non-communicable diseases, and most (57 to 74, 67% to 87%) were incompatible with the Paris Climate Agreement and other environmental targets. In comparison, adoption of the WHO recommendations was associated with similar health and environmental changes, whereas adoption of the EAT-Lancet recommendations was associated with 34% greater reductions in premature mortality, more than three times greater reductions in greenhouse gas emissions, and general attainment of the global health and environmental targets. As an example, the FBDGs of the UK, US, and China were incompatible with the climate change, land use, freshwater, and nitrogen targets, and adopting guidelines in line with the EAT-Lancet recommendation could increase the number of avoided deaths from 78 000 (74 000 to 81 000) to 104 000 (96 000 to 112 000) in the UK, from 480 000 (445 000 to 516 000) to 585 000 (523 000 to 646 000) in the USA, and from 1 149 000 (1 095 000 to 1 204 000) to 1 802 000 (1 664 000 to 1 941 000) in China.

**Conclusions:**

This analysis suggests that national guidelines could be both healthier and more sustainable. Providing clearer advice on limiting in most contexts the consumption of animal source foods, in particular beef and dairy, was found to have the greatest potential for increasing the environmental sustainability of dietary guidelines, whereas increasing the intake of whole grains, fruits and vegetables, nuts and seeds, and legumes, reducing the intake of red and processed meat, and highlighting the importance of attaining balanced energy intake and weight levels were associated with most of the additional health benefits. The health results were based on observational data and assuming a causal relation between dietary risk factors and health outcomes. The certainty of evidence for these relations is mostly graded as moderate in existing meta-analyses.

## Introduction

Our diets connect personal and public health with global environmental sustainability. Imbalanced diets, such as ones low in fruits and vegetables, high in red and processed meat, and providing excessive energy intake, represent one of the greatest health burdens globally and in most regions,^1^ ^2^ and the chronic diseases related to unhealthy diets, such as cardiovascular diseases, cancer, and type 2 diabetes, require costly treatment.^3^ The food system is also a major driver of impacts on the environment,^4^ and without dietary changes towards more plant based diets, key environmental limits related to climate change, land use, freshwater extraction, and biogeochemical flows associated with fertiliser application risk being exceeded.^5^ ^6^ Model based analyses have indicated the potential benefits of dietary changes for reducing environmental resource use, premature mortality from dietary risk factors, and healthcare costs.^7^ ^8^ ^9^

National food based dietary guidelines (FBDGs) are political, government endorsed documents intended to provide context specific recommendations and advice on healthy diets and lifestyles.^10^ Typically they form the basis for educational programmes and national food and nutrition policies in their respective countries.^11^ In addition to impacting the national food environment, FBDGs have important global implications, in particular when consumption patterns are recommended that conflict with attainment of global environmental targets, such as limiting global warming to below 2°C.^12^ ^13^ ^14^ Aligning FBDGs with the latest evidence not just on healthy eating but also on the wider social and environmental implications of dietary choices is therefore an important starting point for enabling policy coherence and building a food environment that contributes to good public and personal health, as well as to local and global environmental sustainability.^5^ ^8^ ^15^

Efforts have been made to compare FBDGs on key messages^15^ ^16^ ^17^ ^18^ ^19^ ^20^ ^21^ ^22^ and to analyse the environmental implications of several FBDGs.^12^ ^13^ ^14^ However, the joint impact on health and sustainability, as well as alignment with global policy targets and established patterns of healthy and sustainable diets have not been analysed comprehensively.

We quantitatively analysed the health and environmental implications of 85 FBDGs around the world. The impacts of the FBDGs were assessed at both the country level and when adopted globally. The effect of universal, global adoption serves as a test for whether the FBDGs are compatible with global challenges and policy targets, including climate change and the associated Paris Agreement on climate change,^23^ mortality from chronic diseases and the Action Agenda on Non-Communicable Diseases,^24^ as well as the sustainable development goals and food related planetary boundaries beyond which ecosystems could be at risk of being destabilised.^5^ ^25^ For providing additional context, we compared the national FBDGs with global dietary recommendations, including those of the World Health Organization^26^ ^27^ and the EAT-Lancet Commission on Healthy Diets from Sustainable Food Systems.^6^

## Methods

Our study followed the guidelines for accurate and transparent health estimates reporting (GATHER) (see appendix for completed checklist). To quantitatively analyse national and global FBDGs, we followed a multistep process (appendix SI figure 1). Firstly, we reviewed existing FBDGs and from the accompanying documents extracted the recommendations for a set of food groups that are relevant for health and environmental impacts. Secondly, we translated the recommendations—some were qualitative and some quantitative—into purely quantitative representations of suggested intake, or change in intake, for each food group. Thirdly, we constructed full diet scenarios by applying the quantitative FBDG recommendations to estimates of current intake for each food group and country. Fourthly, we analysed the potential health and environmental impacts if the populations of countries with FBDGs changed their current diets to those that are in line with their FBDGs as represented by the diet scenarios. Finally, we analysed the alignment of the different FBDGs with global health and environmental targets by modelling the universal, global adoption of each FBDG.

### Coding of dietary guidelines

We used the online repository of FBDGs maintained by the Food and Agriculture Organization of the United Nations to access a country’s national dietary guideline.^28^ The repository summarises existing FBDGs by country and region and provides links to the source documents and a summary of key messages. At the time of our analysis (March to July 2019), the repository listed 97 countries or regions (appendix SI table 1). We obtained the source documents using the links provided, or, when those were broken, through web searches, and we omitted guidelines when we were unable to obtain the source documents in that way. Google Translate (Google LLC, 2019) was used for source documents that were not available in English. We used comparative data provided by the EU Science Hub for reviewing the FBDGs of European countries,^29^ and the dedicated reports published by WHO and the EAT-Lancet Commission for reviewing the global dietary recommendations.^6^ ^26^ ^27^

From each source document we extracted verbatim key messages for 12 food groups that are commonly present in dietary guidelines, and bodyweight (appendix Datafile). The food groups included fruits, vegetables, whole grains, red meat, processed meat, poultry, fish, milk (including dairy products), eggs, legumes, nuts and seeds, and sugar. We included recommendations on balancing energy intake by adjusting the consumption of staple foods, such as grains and potatoes, as a way to increase or decrease energy intake (while maintaining recommendations for whole grains). To analyse adherence to dietary guidelines, we classified the food groups into recommended (fruits, vegetables, whole grains, legumes, nuts and seeds, and fish), discouraged (red meat, processed meat, and sugar), and neutral (milk, eggs, and poultry) based on the associations with diet related disease risk used in the health analysis (appendix SI tables 9 and 10).^30^ ^31^

For translating the dietary recommendations for each food group into a quantitative representation of recommended diets, we developed a coding method, including a score that expressed our degree of confidence in the assigned consumption value (appendix SI table 2):

• The lowest uncertainty (score of 1) was assigned when exact quantities were provided, either as point recommendations (eat five servings of fruits and vegetables a day), range of values (eat 2-3 servings of fruits a day), or qualified values (eat at least five servings of fruits and vegetables a day). When a range of values was provided, we adopted the average as the mean value, and the low and high recommendations as low and high values for use in an uncertainty analysis. Qualified values were adopted as mean estimates, and the mean value was increased or decreased by 20% in the high and low value of the uncertainty analysis, depending on the qualification (eg, at least, or up to).

• When serving sizes were not explicitly defined, we adopted the average serving sizes from FBDGs in the same WHO region (appendix SI table 3) and increased the coding score by 1 (to an uncertainty score of 2).

• Medium uncertainty (score of 3) was assigned for general statements, such as “eat daily” when the serving size is clear (eg, for eggs), or when a value was provided for a more general food group, such as fruits and vegetables instead of fruits. The serving size was unclear, we used a standard serving size (eg, one egg) and coded this as one serving a day. When a value was provided for a more general food group, we used the current distribution across the subgroup (eg, if the split between fruits and vegetable consumption was 1:2, then that was maintained under a recommendation that asked to increase total consumption to five a day).

• High uncertainty (score of 4) was assigned when the recommendations were vague, such as eat regularly, eat multiple times a week, or increase or decrease intake, as well as when recommendations were provided for foods that span categories, such as meat and legumes and all animal products, without providing detail on the relative distribution. We coded recommendations to increase or decrease intake as 20% increase or decrease of current intake, with a range of 10-30% in the low and high values of the uncertainty analysis. Recommendations to eat regularly or multiple times a week were coded when serving sizes were clear (eg, for eggs, nuts and seeds, and legumes) as a range of one serving a day to one serving a week, with the mean value as the average. When daily servings required additional information on quantity (eg, for fruits and vegetables that are commonly consumed more than once a day), then these recommendations were not coded. Recommendations that spanned different food categories were coded by proportionally assigning the recommended value to each mentioned food group (eg, recommendations to consume four protein foods a day, including meat, legumes, nuts, and eggs were coded as one serving each a day (four servings over four categories) of meat, legumes, nuts, and eggs).

• Recommendations that were vaguer than those mentioned previously (eg, eat fruits and vegetables) were not coded and were assigned the highest uncertainty (score of 5).

Recommendations related to bodyweight were often vague or provided in documents other than FBDGs. We therefore coded recommendations for bodyweight on a binary scale (yes or no). A yes answer was assigned if the FBDG recommended attainment of a healthy bodyweight—for example, by balancing energy intake with physical activity or by regulating energy intake.

### Construction of guideline scenarios

We used the coded FBDG values to construct consumption patterns that if consumed by a representative consumer in the relevant country would meet the FBDG recommendations. For that purpose, we applied the coded values (some of which were expressed in relation to baseline intake, such as 20% (10-30) increase in consumption of fruits and vegetables) to estimates of baseline intake by country and food group. If FBDGs included recommendations to attain a healthy weight, we adjusted the intake of staple foods (grains and roots) to attain an average energy intake at population level that was in line with estimates of optimal body mass index (BMI) levels for each country’s population structure (appendix SI tables 5 and 6).^8^ If no recommendation was provided, we assigned baseline intake.

Because regionally comparable data on full diets do not exist at present, we derived our own proxies. We used globally comparable estimates of the amount of food that is available for consumption in a country, provided by the Food and Agriculture Organization, and adjusted the estimates for food wasted during consumption (appendix SI tables 7 and 8).^32^ ^33^ To estimate the intake of whole grains and processed meat, we applied processing ratios derived from survey data^34^ to the baseline estimates (appendix information SI.2). An alternative would have been to rely on a set of consumption estimates that has been based on a variety of data sources, including dietary surveys, household budget and expenditure surveys, and food availability data.^34^ ^35^ However, neither the exact combination of these data sources nor the estimation model used to derive the data have been made publicly available. For some countries, using dietary surveys would also have been an alternative. Underreporting is, however, a persistent problem in dietary surveys,^36^ ^37^ and regional differences in survey methods would have meant that our results would not be comparable between countries.

We developed three criteria for excluding FBDGs that lacked sufficient data for a quantitative evaluation. For the first criterion we scored whether the full FBDG could be accessed and understood by the author after translation. The second criterion was whether recommendations were provided for either fruits and vegetables or red meat (two of the major food groups relevant for the health and environmental impacts of diets), and, additionally, whether the overall uncertainty score, calculated as the average of the coding scores of the individual food groups, was above 4.5. The third criterion was whether baseline consumption data were available. Based on these criteria, we excluded 11 FBDGs; nine related to the first two criteria and two because of a lack of baseline data (appendix SI figure 2). After exclusions, 86 national and two global FBDGs were left for further analysis. As Belgium had two FBDGs for different regions, we took the average of the quantitative representation of both. The appendix Datafile provides the coding and uncertainty scores for the 97 countries reviewed for this study.

### Health analysis

Using an established modelling framework, we analysed the health and environmental impacts of dietary changes to diets that conform to FBDGs.^8^ To analyse the health implications of adopting FBDGs, we used a comparative risk assessment framework to estimate changes in deaths from non-communicable diseases. Our analysis covered 11 risk factors and five disease endpoints. The risk factors included high consumption of unprocessed red meat and processed meats, low consumption of fruits, vegetables, nuts and seeds, whole grains, fish, and legumes, and being underweight (BMI <18.5), overweight (25 <BMI <30), or obese (BMI >30). The disease endpoints included coronary heart disease, stroke, type 2 diabetes mellitus, cancer (in aggregate and as colon and rectum cancers), and respiratory disease (which is associated with changes in weight).

The disease endpoints accounted for about half of the deaths in 2015,^38^ and the risk factors were responsible for two thirds of deaths attributable to dietary risk factors in 2015 and for a third of all attributable deaths in that year.^39^ In low income settings, the adoption of healthy diets, and in particular balanced energy intake, would have additional impacts on reducing acute forms of malnutrition.^40^ Because we do not explicitly capture these impacts, our estimates can be considered conservative, in particular for low income countries.

We estimated the mortality and disease burden attributable to dietary risk factors by calculating population impact fractions^41^ and applying those to age and country specific mortality rates.^38^ Population impact fractions represent the proportions of disease cases that would be avoided when the risk was changed from a baseline situation (the baseline diets) to a counterfactual situation (the dietary guideline scenarios). Relative risk estimates that relate risk factors to disease endpoints were adopted from meta-analyses of prospective cohort studies (appendix SI table 9).^42^ ^43^ ^44^ ^45^ ^46^ ^47^ ^48^ ^49^ ^50^ In line with the meta-analyses, we included non-linear dose-response relations for fruits and vegetables, nuts and seeds, whole grains, and fish, and assumed linear dose-response relations for the remaining risk factors. As our analysis was primarily focused on mortality from chronic diseases, we focused on adults aged 20 or older, and we adjusted the relative risk estimates for attenuation with age based on a pooled analysis of cohort studies focused on metabolic risk factors,^51^ in line with other assessments.^38^

### Environmental analysis

To analyse the environmental implications of adopting FBDGs, we used country and crop specific environmental footprints for greenhouse gas emissions, cropland use, freshwater use, and nitrogen and phosphorus application (appendix SI table 11).^5^ The footprints are based on global datasets on environmental resource use in the producing region^52^ ^53^ ^54^ ^55^ and have been adjusted for the proportion of food, and the associated footprint, that is imported, exported, and processed to reflect the resource demand of consuming a specific food in a specific country.^5^ ^8^

The footprints for greenhouse gas emissions include methane and nitrous oxide but exclude carbon-dioxide, most of which, following the methodology of the International Panel on Climate Change, are allocated to the energy, transport, and processing sectors.^54^ ^56^ ^57^ The land footprints focus on the demand for cropland in line with previous assessments and therefore do not include pastures.^5^ The freshwater footprints account for the consumptive use of surface water and groundwater,^52^ and the nitrogen and phosphorus footprints account for application from fertiliser use.^55^ The footprints for oils and sugar account for country specific processing factors, and those for animal source foods account for the environmental impacts associated with feed production, with country specific feed efficiencies and compositions.^52^

### Health and environmental targets

We analysed the health and environmental impacts of adopting FBDGs at both a national level and a global level. The global analysis was intended as a test to determine whether a FBDG is compatible with global health and environmental targets that are associated with diets. The targets included the sustainable development goal of reducing premature mortality from non-communicable diseases by a third, the Paris Agreement to limit global warming to below 2°C, the Aichi biodiversity target of limiting the rate of land use change, as well as the sustainable development goals and planetary boundaries related to freshwater use, and nitrogen and phosphorus pollution (appendix SI table 12).

We analysed the compatibility of the FBDGs with the targets by modelling the universal global adoption of each FBDG (appendix information SI.5). For that purpose, we changed the baseline intake of 169 countries (all countries for which we were able to obtain consumption, health, and environmental data) to each of the different FBDG diet scenarios in turn, and assessed global health and environmental impacts. We then compared the global health and environmental impacts to the diet related portion of the different health and environmental targets,^5^ such as the emissions budget allocated to food production under a climate stabilisation pathway that is in line with fulfilling the Paris Agreement,^58^ or what proportion of non-communicable disease risks are due to dietary risks.^2^ When targets were expressed for future years, we used projections of environmental footprints that included improvements in technologies and management practices (eg, implementation of agricultural mitigation options and improvements in crop yields, irrigation, and fertiliser application) along a middle-of-the-road socioeconomic development pathway.^5^

### Uncertainty analysis

We captured uncertainty in several ways. In constructing the FBDG scenarios, we accounted for the uncertainty related to interpreting the recommendations of the different FBDGs by assigning uncertainty scores to each FBDG and by adopting high and low values of the recommendations in a dedicated uncertainty analysis. In the health analysis, we accounted for the uncertainty of the risk-disease associations by estimating standard deviations around the mean estimate using standard methods of error propagation. In the environmental analysis, we accounted for the uncertainty of the global targets by deriving upper and lower values, which we considered in a dedicated uncertainty analysis.

### Patient and public involvement

The modelling study was based on population averages, which was not conducive to involving members of the public in the study conception, design, data analysis, or reporting. The public was not included in advisory or consultation roles and was not invited to comment on the paper before submission.

## Results

**Fig 1:**
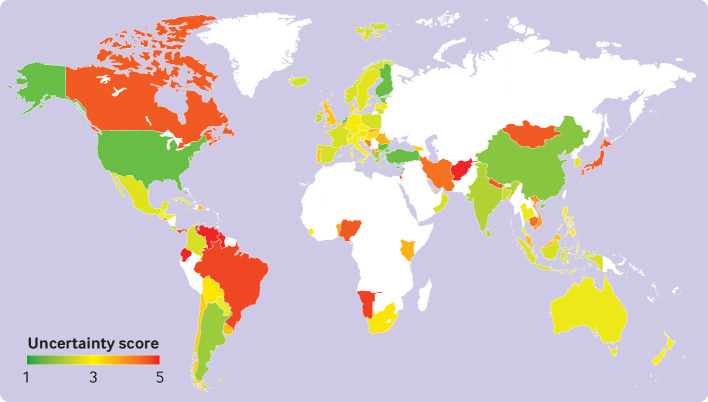
Overview of countries with food based dietary guidelines (FBDGs) and the average uncertainty score of each FBDG. Uncertainty was coded on a scale of 1 (low uncertainty) to 5 (high uncertainty) and averaged across recommendations for fruits and vegetables, legumes, nuts and seeds, whole grains, milk, eggs, fish, sugar, red meat, and processed meat. Appendix SI table 13 lists the uncertainty scores by food group

Overall, 99 FBDGs (97 national and two global) were reviewed and the uncertainty of the FBDG recommendations scored for 12 food groups and bodyweight (fig 1). After excluding FBDGs for countries that did not report food availability data to the FAO and that were either inaccessible or attained high uncertainty scores because they contained little concrete recommendations, 85 national FBDGs and two global sets of dietary recommendations remained for further analysis (appendix SI figure 2). We used the geographical regions to accord with those of the Food and Agriculture Organization of the United Nations (appendix SI table 1),^59^ which, for example, uses Near East to include Iran, Lebanon, Oman, and Qatar. Out of the 85 national FBDGs, 36 (42%) were from Europe, 23 (27%) from Latin America and the Caribbean, 15 (18%) from Asia and the Pacific, 6 (7%) from Africa, 3 (4%) from the Near East and 2 (2%) from North America.

### Uncertainty scores

The level of uncertainty associated with the dietary recommendations differed considerably across the FBDGs and food groups. Among national FBDGs (fig 1), the region with the clearest guidelines and that therefore attained the lowest uncertainty score was the WHO Europe region (uncertainty score 2.9, on a scale of 1 for low uncertainty to 5 for high uncertainty), followed by the Near East (3.0), North America (3.0), Asia and the Pacific (3.3), Latin America and the Caribbean (3.4), and Africa (3.8). Across food groups (appendix SI table 13), the clearest guidance was for fruits and vegetables (1.9), followed by milk (2.3), sugar (2.8), fish (2.9), legumes (3.2), eggs (3.3), red meat (3.4), nuts and seeds (3.8), whole grains (3.9), and processed meat (4.3). Most FBDGs, especially those in North America and the Near East, and 82% overall, included advice to balance energy intake to avoid overweight and obesity.

The global recommendations diverged on uncertainty scores and attained both lower and higher scores than the national FBDGs (appendix SI table 13). The WHO recommendations included a limited number of clear recommendations (on fruit and vegetable intake, sugar, and energy balance) and therefore attained a relatively high uncertainty score (4.0), whereas the EAT-Lancet recommendations included recommendations for all major food groups and energy intake and therefore attained a low uncertainty score (1.0).

### Dietary recommendations

**Fig 2:**
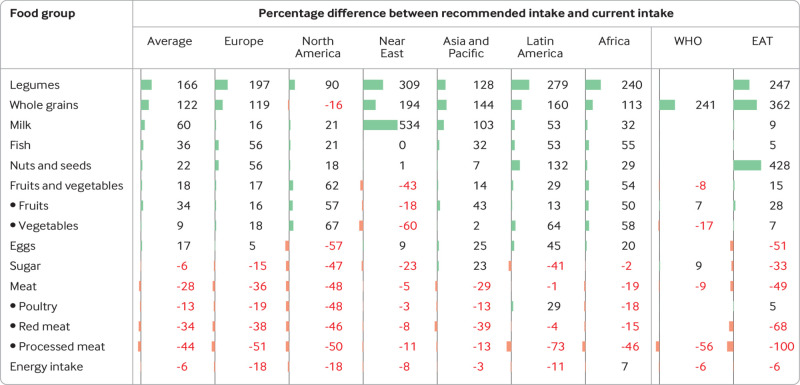
Percentage difference between recommendations from food based dietary guidelines (FBDGs) and current intake by food group and region. Positive values (in black) indicate greater intake in FBDGs and negative ones (in red) indicate lower intake. The comparison is based on recommended mean values. For the global FBDGs, the percentage changes between the guidelines and current intake is the average across all countries with a FBDG. WHO=World Health Organization; EAT=EAT-Lancet Commission on Healthy Diets from Sustainable Food Systems

Compared with current intake (fig 2), the representative consumption patterns adhering to the mean values of the national FBDGs included, on average, more fruits and vegetables (18%, range across regions 14% to 62%, except for the Near East, where high consumption levels already exceed recommendations), legumes (166%, 90% to 309%), nuts and seeds (22%, 1% to132%), whole grains (122%, 113% to 194%, except for North America), milk (60%, 16% to 534%), eggs (17%, 5% to 45%, except for North America), and fish (36%, 0% to 56%), less sugar (−6%, −2 to −47%, except for Asia and the Pacific) and meat (−28%, −1% to −48%), especially red and processed meat (−34%, −4% to −46%; −44%, −11% to −73%, respectively), and an energy intake that was lower on average than current intake (−6%, −3% to −18%), except for Africa where current energy intake was below recommendations.

Compared with the consumption patterns that adhered to the national FBDGs, those that adhered to the mean values of the WHO recommendations included less fruits and vegetables (−8% *v* 18%), less processed meat (−56% *v* −44%), more whole grains (241% *v* 122%), more meat (−9% *v* −28%), and more sugar (9% *v* −6%), and similar energy intake (−6%), whereas those adhering to the mean values of the EAT-Lancet recommendations included similar amounts of fruits and vegetables (15% *v* 18%) and similar energy intake (−6%), but more legumes (247% *v* 166%), nuts and seeds (428% *v* 22%), and whole grains (362% *v* 122%), and less milk (9% *v* 60%), sugar (−33% *v* −6%), and meat (−49% *v* −28%), especially red and processed meat (−68% and −100% *v* −34% and −44%, respectively), and fewer eggs (−51% *v* 17%).

**Fig 3:**
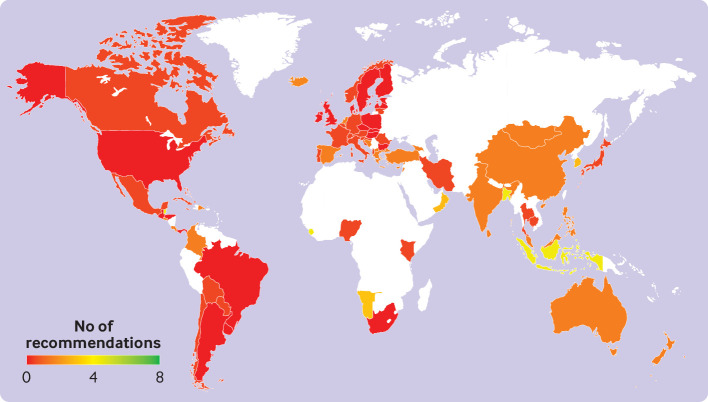
Number of food based dietary guidelines (FBDGs) recommendations that were achieved in each country. The number of recommendations included increases in fruits and vegetables, legumes, nuts and seeds, whole grains, and fish, as well as reductions in sugar and red meat and processed meat. The comparison is based on recommended mean values

Although current consumption patterns fulfilled some aspects of the FBDGs, no country simultaneously fulfilled all recommendations for the food groups that are considered recommended (fruits and vegetables, legumes, nuts and seeds, whole grains, fish) or discouraged (sugar, red meat, processed meat) (fig 3). Of the 85 countries with national FBDGs, more than a quarter (28%, n=24) met no recommendation, 88% (n=75) met no more than two recommendations, and no country simultaneously fulfilled five, six, seven, or all eight recommendations. The countries that each fulfilled four recommendations included Bangladesh (fish, sugar, red meat, processed meat), Indonesia (sugar, red meat, processed meat, whole grains), and Sierra Leone (fish, sugar, red meat, whole grains). Across regions (appendix SI figure 3), Asia and the Pacific had relatively high attainment for fish (60%), Africa for whole grains and sugar (50% each), and the Near East for fruits and vegetables (100%). Within North America, the United States did not attain any recommendation, whereas Canada only fulfilled those for whole grains.

### Regional health impacts

**Fig 4:**
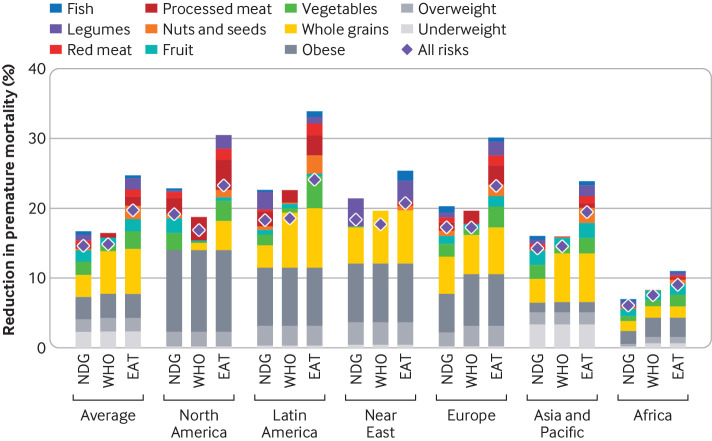
Reduction in premature mortality (among ages 30-70) by region, scenario, and risk factor. The scenarios include adoption of national food based dietary guidelines (NDG), World Health Organization recommendations (WHO), and the EAT-Lancet Commission on Healthy Diets from Sustainable Food Systems recommendations (EAT). Risk factors include reductions in intake of fruits, vegetables, legumes, nuts and seeds, whole grains, and fish, increases in intake of red and processed meats, and increases in the prevalence of underweight, overweight, and obesity. The health impacts associated with the combination of all risks is smaller than the sum of individual risks, because the former controls for coexposure (that is, each death is attributed to one risk factor only)

Adoption of national FBDGs and without reducing recommended food groups or increasing discouraged ones was associated with a reduced burden from diet related, non-communicable diseases, with reductions in premature mortality of 15% (13% to 16%) on average (fig 4; appendix SI figure 4). About 43% of the reductions were from improved weight levels, in particular reduced prevalence of obesity (19% of overall reductions), overweight (11%), and underweight (13%). Changes in food composition were responsible for the remaining reductions in mortality, in particular increased intake of whole grains (19%), vegetables (11%), fruits (10%), legumes (5%), fish (3%), and nuts and seeds (1%), and reduced intake of processed and red meats (4% and 3%, respectively). Across regions, the reductions in mortality ranged from 6% in Africa, where much of the health burden is still associated with communicable diseases, to 19% in North America, where reductions in the high prevalence of obesity in that region contributed to large reductions in mortality. At the country level, the reductions in premature mortality ranged from 4% for Nigeria to 30% for Bulgaria (appendix SI figure 5).

Compared with the national FBDGs, adoption of the global recommendations was associated with reductions in premature mortality, which on average were similar for the WHO recommendations but 34% greater for the EAT-Lancet recommendations (fig 4). For the WHO recommendations, lower benefits associated with less ambitious recommendations on fruit and vegetable intake and the lack of recommendations for many other dietary risks were compensated by greater benefits associated with recommendations for whole grains and, in some regions, bodyweight. For the EAT-Lancet recommendations, most of the additional reductions in premature mortality stemmed from more ambitious recommendations for the intake of whole grains, nuts and seeds, legumes, processed meat, and vegetables. Across regions, the WHO recommendations were associated with greater benefits relative to national FBDGs in Africa (22%), where national FBDGs were relatively less developed, and with less benefits in North America (−12%), where national FBDGs were relatively comprehensive. In comparison, the EAT-Lancet recommendations were associated with additional benefits in all regions, ranging from 12% in the Near East to 47% in Africa (appendix SI figure 5).

### Regional environmental impacts

**Fig 5:**
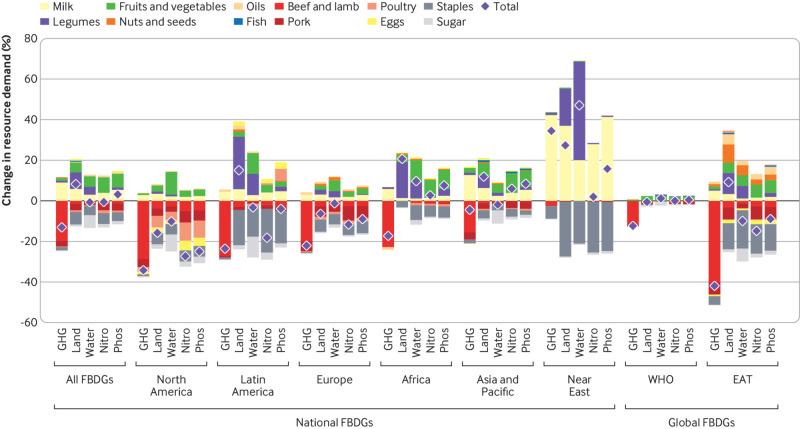
Change in environmental resource demand for adopting national or global (World Health Organization (WHO), EAT-Lancet Commission on Healthy Diets from Sustainable Food Systems (EAT)) food based dietary guidelines (FBDGs) by food group and environmental domain. The environmental domains include food related greenhouse gas emissions (GHG), cropland demand (land), freshwater demand (water), nitrogen demand from fertilisers (nitro), and phosphorus demand from fertilisers (phos)

Adoption of national FBDGs led to changes in environmental impacts and resource demand (fig 5; appendix SI figure 6). Food related greenhouse gas emissions were reduced on average by 13% (550 mega tonnes of carbon dioxide equivalent) across all countries with national FBDGs, most of which were driven by reductions in consumption of ruminant meat and were offset—in part in most regions, but in full in the Near East—by increased milk consumption. Demand for cropland increased on average by 8% (590 million square kilometres), driven by increases in the consumption of milk, legumes, and fruits and vegetables, in particular in the Near East, Latin America and the Caribbean, Asia and the Pacific, and Africa, but increases were compensated by reductions in the consumption of animal products and staple crops in Europe and North America. Demand for freshwater stayed similar (−0.4%, −5 km^3^), as increased demand for fruits and vegetables, legumes, and milk was compensated by less demand for sugar, staples, and animal products in most regions, except for Africa and the Near East. The demand for nitrogen and phosphorus also stayed similar in total (−0.2%, 160 giga grams; 3%, 365 giga grams), as increased demand from fruits and vegetables and from milk was offset—in part in Asia and the Pacific, Africa, and the Near East, and in full in Europe, North America, and Latin America and the Caribbean—by reduced demand for staple crops, animal products, and sugar.

Compared with the national FBDGs, adoption of the global recommendations resulted in moderate reductions in resource demand when adopting the WHO recommendations, and in greater changes when adopting the EAT-Lancet recommendations (fig 5). Adoption of the WHO recommendations was associated with a reduction in greenhouse gas emissions on average by 12% (520 million tonnes of carbon dioxide equivalent) in countries with national FBDGs, in particular as a result of lower consumption of ruminant meat (associated with the recommendations on processed meat). On other domains, small increases in resource demand from greater fruit and vegetable consumption were compensated by reductions from less staple crops (associated with reductions in overweight and obesity) and from less processed meat and sugar. By comparison, adoption of the EAT-Lancet recommendations was associated with large net reductions in greenhouse gas emissions (−42%, −1.8 giga tonnes of carbon dioxide equivalent), freshwater use (−10%, −110 km^3^), and nitrogen and phosphorus application (15%, 9.6 tera grams; 9%, 1.0 tera grams), and to a similar increase in cropland (9%, 670 million square kilometres) as the national FBDGs. Most of the reductions in greenhouse gas emissions were associated with stricter limits on red meat, and for the other environmental domains, the reductions from less animal products, staple crops, and sugar, exceeded the increases associated with more fruits, vegetables, nuts and seeds, legumes, and oils.

### Global health and environmental impacts

**Fig 6:**
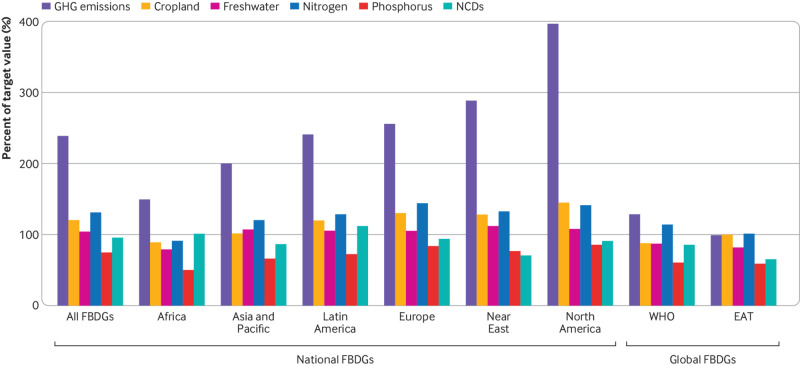
Comparison of the health and environmental impacts of universally adopting food based dietary guidelines (FBDGs) to a set of global health and environmental targets. Targets include the sustainable development goal of reducing premature mortality from non-communicable diseases (NCDs) by a third, the Paris Climate Agreement to limit global warming to below 2°C (greenhouse gas (GHG) emissions), the Aichi biodiversity target of limiting the rate of land use change (cropland), and the sustainable development goals and planetary boundaries related to freshwater use, and nitrogen and phosphorus pollution. Estimates are expressed as percentage of attained target value averaged across countries in FBDG regions. Values of 100% or less indicate that environmental and health impacts are in compliance with the targets, and values greater than 100% indicate that targets are exceeded. WHO=World Health Organization; EAT=EAT-Lancet Commission on Healthy Diets from Sustainable Food Systems

Modelling the universal adoption of the FBDGs along a middle-of-the-road development trajectory to 2050 indicated potential mismatches between most FBDGs and global environmental and health targets. The greatest mismatch concerned the food related emissions targets compatible with the Paris Agreement, which were exceeded by 140% on average, ranging from 50% for the African FBDGs to 300% for those in North America (fig 6), where meat and dairy consumption remained high despite relative reductions (appendix SI figures 7 and 8). At the country level, of the 85 national FBDGs, 56 (66%) met the diet related target for non-communicable diseases of reducing premature mortality from such diseases by a third, 11 (13%) were compatible with a food related emissions pathway of limiting global warming to below 2°C in line with the Paris Agreement, 19 (22%) were in line with global land use targets, 28 (33%) were in line with freshwater targets, 9 (11%) fulfilled nitrogen targets, and all were in line with phosphorus targets, when combined with technological improvements and reductions in food loss and waste (appendix SI figures 9a-f).

**Fig 7:**
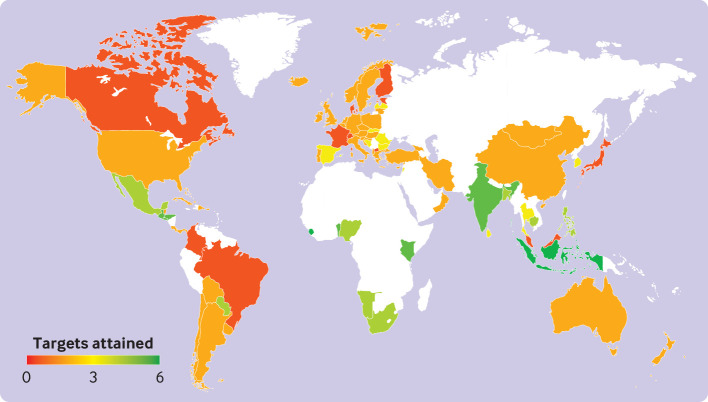
Number of global health and environmental targets attained by country. Targets include the sustainable development goal of reducing premature mortality from non-communicable diseases by a third, Paris Agreement to limit global warming to below 2°C, Aichi biodiversity target of limiting the rate of land use change, and sustainable development goals and planetary boundaries related to freshwater use, and nitrogen and phosphorus pollution

Summed across targets, about two thirds of the national FBDGs fulfilled only one (n=17, 20%) or two (n=40, 47%) of the six global health and environmental targets, 11 (13%) fulfilled three, 9 (11%) fulfilled four, 6 (7%) fulfilled five, and 2 (2%) fulfilled six (fig 7). The national FBDGs that fulfilled all six targets were those of Indonesia and Sierra Leone. The FBDGs of these countries have in common recommendations for a low to modest intake of meat and dairy relative to their current consumption patterns. Indonesia recommends some meat intake, but it does not provide a quantitative recommendation for milk, which was coded as no change to their low baseline levels, and Sierra Leone recommends a serving of one animal product a day, and no change to its low baseline consumption of milk.

The EAT-Lancet recommendations contain similarly low amounts of meat and dairy but also include explicit recommendations for all other major food groups that when universally adopted were associated with reductions in premature mortality and environmental resource demand in line with the full set of global health and environmental targets (fig 6). In comparison, universal adoption of the WHO recommendations fulfilled the global target for non-communicable diseases, but it fell 29% short of the climate change target, 14% short of the nitrogen target, and, similar to baseline diets, it fulfilled the cropland, freshwater, and phosphorus targets, provided resource efficiency increases as projected. The global impacts of the WHO and EAT-Lancet recommendations were dominated by low resource demand in countries without national FBDGs, most of which are in low and middle income countries (appendix SI table 17). Calculating the average global impacts only across countries with national FBDGs showed that the WHO recommendations adopted in those countries exceeded the target for greenhouse gas emissions by 165% and that for nitrogen by 38% (appendix SI figure 8). The difference in impacts was less pronounced for the EAT-Lancet recommendations owing to the more comprehensive coverage of food groups.

### Uncertainty analysis

To analyse the uncertainty of interpreting the different FBDGs, the low and high values of the recommendations for each food group were used to construct FBDG representations that included more of the recommended foods and less of the discouraged and neutral ones, and vice versa (appendix information SI.7). Compared with using the mean values of the recommendations, the representation with greater portions of recommended foods had 11-45% more fruits, vegetables, legumes, nuts, whole grains, and fish, and 3-22% less red meat, sugar, milk, and eggs, whereas the representation with greater portions of discouraged foods had 5-23% less and 1-32% more of those foods, respectively. The reductions in premature mortality increased by 25% in the former and decreased by 15% in the latter; greenhouse gas emissions were reduced by 6% in the former and increased by 31% in the latter; and the demand for other environmental resources showed little difference (appendix SI table 14). Doing the same for the WHO recommendations showed either an increase in the reductions in premature mortality by 15% or a decrease by 4% compared with the mean values of the recommendations, and greenhouse gas emissions changed by 7% in either direction.

For analysing the uncertainty of interpreting the EAT-Lancet recommendations, different dietary patterns were constructed that are in the line with the general recommendations, including pescatarian diets that contain no meat and relatively more fish and seafood, vegetarian diets that contain no meat or fish but more legumes and fruits and vegetables, and vegan diets that contain no animal source foods but more legumes and fruits and vegetables. The different dietary patterns were associated with 4-14% greater reductions in premature mortality compared with the standard EAT-Lancet recommendations and were associated with 39-69% greater reductions in greenhouse gas emissions and moderate changes in other resource use, in each case with greatest impacts for adoption of vegan diets (appendix SI table 15).

For analysing the uncertainty related to the global health and environmental targets, the health and environmental impacts were compared with the low, mean, and high values of the target’s uncertainty range (appendix SI table 12). Most of the national FBDGs attained one to two mean value targets (57, 67%), three to four high value targets (55, 65%), and zero to one low value targets (78, 92%). Whereas two FBDGs fulfilled all mean value targets, 11 fulfilled all high value targets, but none fulfilled all low value targets (appendix SI figure 10).

## Discussion

Current diets in most countries are neither healthy nor environmentally sustainable. In the current study we show that dietary changes towards those recommended by national FBDGs could be associated with reductions in premature mortality, in particular from non-communicable diseases, in all of the 85 countries with FBDGs that were included in the analysis. The environmental implications of such changes were, however, mixed. Although adoption of some FBDGs was associated with reductions in greenhouse gas emissions and, for a small number of FBDGs, with reductions in other environmental resource demand, the changes were generally moderate, and most FBDGs were not compatible with a set of global environmental targets, including the Paris Agreement, the Aichi biodiversity targets related to land use, and the sustainable development goals and planetary boundaries related to water use and fertiliser application. By comparison, following the dietary recommendations issued by the WHO was in most cases associated with similar health benefits and changes in environmental resource demand, whereas adopting a set of dietary recommendations developed by the EAT-Lancet Commission on Healthy Diets from Sustainable Food Systems with the intention to merge health and sustainability aspects was associated with greater health benefits than the national FBDGs, and a reduction in environmental resource use in line with global environmental targets.

Our results suggest that reforming national FBDGs, as well as WHO guidelines, could be not only beneficial from a health perspective but also necessary for meeting global sustainability goals and staying within the environmental limits of the food system. From an environmental perspective, the most important aspects that differentiated current FBDGs from dietary patterns that stayed within environmental limits were the amounts of animal source foods, in particular red meat and dairy. Whereas many national FBDGs recommended some reductions in red meat intake, most were much less ambitious than the EAT-Lancet recommendations, which suggest to limit the consumption of red meat to one serving a week based on the association between red meat and increased risk for mortality from non-communicable diseases.^6^ The greater reduction in red meat intake was associated with most of the reductions in greenhouse gas emissions, and in turn the diets were compatible with the climate change target of limiting global warming to below 2°C. The reductions in red meat were also associated with more than a third of the reductions in cropland demand and nitrogen and phosphorus application and for more than 10% of the reductions in freshwater demand (fig 5).

Most national FBDGs recommended increasing dairy consumption relative to current diets, which resulted in substantial increases in environmental impacts across all environmental dimensions. In contrast, the EAT-Lancet recommendations suggest limiting dairy intake to one serving or glass a day, based on the absence of a clear association between milk intake, bone health, and reduced risk of non-communicable diseases,^8^ and the existence of plant based alternatives that have similar nutrient content and are more clearly associated with reduced risks.^30^ The recommendation was associated with reduced dairy consumption and lower environmental impacts in many high income countries and avoided what according to national FBDGs and projected trends would be increasing consumption and environmental impacts in middle and low income countries. According to our analysis, more than three quarters of the increases in greenhouse gas emissions when national FBDGs were adopted were from dairy (which, in net, were compensated by reductions in red meat intake), and a quarter to more than a third of the increases in the other environmental domains (fig 5). Avoiding these increases and reducing intake in high consuming countries made a major contribution to staying within the global environmental targets.

Other factors were also important for both health and the environment. One important factor, in particular for fulfilling the land, water, and fertiliser targets, was the reduction in excessive energy intake that was associated with more ambitious targets on sugar intake, and reduced consumption of staple crops such as grains and potatoes. The reductions in sugar and staples were associated with over half of the reductions in the demand for land, water, and fertilisers (fig 5). Reducing excessive energy intake and, through that, the proportion of overweight and obesity was also one of the most important aspects for reducing premature mortality from non-communicable diseases, contributing a third to the overall reductions achieved by adopting the EAT-Lancet recommendations (fig 4). Other factors important for health were the recommended increases in whole grains, fruit and vegetables, nuts and seeds, and legumes. Especially the recommendations on plant based protein sources, such as legumes and nuts and seeds, were either lacking in many national FBDGs or relatively vague. A previous global review showed that less than a half of national FBDGs (44%) depict both plant and animal sources of protein together in the same “protein” food category, and most countries (81%) have no key message about nuts and seeds.^16^ Providing explicit targets for plant based protein sources and providing a clear link to lower limits for animal products when current and projected intake is too high would bring national FBDGs more in line with healthy and environmentally sustainable dietary patterns.

### Strengths and limitations of this study

We carried out a quantitative assessment of most of the existing FBDGs against a comprehensive set of health and environmental indicators. Previous studies have either compared FBDGs on key messages without attempting a quantitative analysis,^15^ ^16^ ^17^ ^18^ ^19^ ^20^ ^21^ ^22^ or quantitatively analysed some of the environmental implications of a few FBDGs.^12^ ^13^ ^14^ Our analysis is most similar to those latter studies. In contrast to those, however, we developed a consistent coding method to translate the mostly qualitative recommendations of the FBDGs into quantitative ones, and we jointly analysed the health and environmental implications of dietary changes towards the FBDGs by country and globally. We also made sure to construct plausible diets based on the FBDGs, something that was not always done in the more environmentally focused FBDG literature.^13^ ^14^ Our analysis was comprehensive in terms of regional coverage and the indicators included. We doubled the number of FBDGs that were analysed in previous studies,^14^ and we extended the use of global sustainability tests for analysing the implications of universally adopting FBDGs from a focus on greenhouse gas emissions^7^ ^13^ to a broad set of health and environmental indicators. Finally, we contrasted the set of national FBDGs with existing global recommendations and previously unavailable recommendations on healthy and sustainable eating, which allowed us to provide specific guidance on FBDG reform.

As with any study, our analysis is subject to several limitations, and there are many potential implications for improvement in future studies. In the health analysis, we used relative risk factors that are subject to the weaknesses common in nutritional epidemiology, including small effect sizes and potential measurement error of dietary exposure, such as overreporting and underreporting and infrequent assessment.^60^ For our calculations, we assumed that the risk-disease relations describe causal associations, an assumption supported by the existence of statistically significant dose-response relations in meta-analyses, the existence of plausible biological pathways, and supporting evidence from experiments, such as on intermediate risk factors.^46^ ^47^ ^48^ ^49^ ^50^ ^61^ ^62^ ^63^ ^64^ ^65^ ^66^ However, residual confounding with unaccounted risk factors cannot be ruled out in epidemiological studies. To address residual confounding, we omitted risk-disease associations that became non-significant in fully adjusted models, in particular those related to milk intake,^67^ ^68^ but potential confounding might also exist for the association between increased fish intake and reduced risk of coronary heart disease.^69^ ^70^ ^71^ ^72^ The quality of evidence in meta-analyses that covered the same risk-disease associations as used here was graded with NutriGrade (grading of recommendations assessment, Development and Evaluation (GRADE) tailored to nutrition research) as moderate or high for all risk-disease pairs included in the analysis (appendix SI table 10).^46^ ^47^ ^61^ In addition, the Nutrition and Chronic Diseases Expert Group graded the evidence for a causal association of 10 of the 14 cardiometabolic risk associations included in the analysis as probable or convincing,^65^ and the World Cancer Research Fund graded all five of the cancer associations as probable or convincing.^73^ The relative health ranking of leading risk factors found in our analysis was similar to existing rankings that relied on different relative risk variables and exposure data.^1^ ^74^

In our analysis, we focused on those food groups that were sufficiently represented in national FBDGs and for which we had robust enough data to provide a quantitative analysis. As a result, several food groups with significant health or environmental associations were omitted. On the health side, those included recommendations to increase polyunsaturated fatty acids in place of saturated fats and to moderate sodium intake, both of which would confer additional health benefits (see appendix SI table 16 for a sensitivity analysis on fat intake).^1^ ^75^ ^76^ On the environmental side, recommendations to moderate the intake of food products that have been associated with deforestation, such as cocoa and other stimulants, could lower pressures on land use and biodiversity loss.^4^ On the other hand, providing recommendations about the geographical origin of foods (regional versus global), as some national FBDGs in Nordic countries do, is unlikely to improve environmental resource use considerably, and especially not greenhouse gas emissions given the relatively low contribution of transport related emissions (estimated to be around 5% to food related emissions in total).^4^

One of the biggest difficulties we encountered during the study was the translation of mostly qualitative guidelines provided by the various FBDGs into quantitative recommendations. To communicate this uncertainty, we developed an uncertainty score and plausible ranges for each food group. Analysis of those ranges indicated substantial uncertainty in the quantitative representation of many FBDGs (appendix SI table 14). Likewise, the uncertainty scores highlight the need for improving the detail of the guidance provided (fig 1). Recommendations were most concrete for fruits and vegetables, milk, and sugar, and often (but not always) more specific in higher income countries compared with lower income ones. Vague recommendations are not only a problem for any quantitative analyses of FBDGs, but they also risk not being understood well by the general public.

Providing general recommendations and specific examples, including exemplary dietary patterns, could help improve the degree to which FBDGs are understood. Our main analysis included one representation of a healthy and sustainable diet, but the sustainable diet literature indicates that various dietary patterns are compatible with good health and global environmental targets.^7^ ^9^ ^77^ In addition to the predominantly plant based flexitarian dietary pattern recommended by the EAT-Lancet Commission, those include mostly plant based pescatarian diets based on sustainable aquaculture, vegetarian diets that include moderate amounts of dairy and eggs, and completely plant based, vegan diets that are based on a variety of fruits and vegetables, whole grains, and plant based protein sources, such as legumes and nuts.^6^ ^8^ The sensitivity analysis indicated that adoption of these dietary patterns might lead to further reductions in premature mortality and environmental resource use, in particular greenhouse gas emissions (appendix SI table 15). Illustrating the breadth of healthy and sustainable diets with reference to the latest understanding of health and environmental implications would represent a big improvement over many current FBDGs and help people navigate an increasingly complex food environment.

More than half of all countries have no national FBDGs, or did not register them with the Food and Agriculture Organization. Our global sustainability tests indicated the large potential that lies in working with countries that currently do not have their own FBDGs (appendix SI tables 17 and 18), many of which are low or middle income countries with diets projected to change towards Western diets as income increases, which would generate additional pressures on the health system and the environment.^5^ ^8^ For countries without national FBDGs, the general dietary recommendations provided by WHO and the procedural recommendations by the Food and Agriculture Organization would often be the starting points for developing national guidelines. Our analysis shows that the current WHO recommendations lack sufficient detail to optimise health and reduce environmental impacts in line with sustainability goals, and a sensitivity analysis showed that the existing recommendations on red and processed meats, such as those by the World Cancer Research Fund, are not ambitious enough either (appendix SI table 15). For improving global dietary recommendations, a more comprehensive and specific set of recommendations would be necessary, including suggested minimum values for whole grains, fruits and vegetables, nuts, and legumes, and more ambitious limits for red and processed meats and dairy. Another important aspect for developing evidence based FBDGs, but one that is rarely adhered to, is the use of accepted methods for reviewing the underlying evidence, rating its quality, and grading the recommendations,^78^ as well as reflecting on the methodological quality of the development process.^79^

### Policy implications

The development of FBDGs that are healthy and sustainable is an important starting point for encouraging the uptake of healthy and sustainable diets at a population level. However, our analysis also showed that less than half of all countries with national FBDGs fulfilled any of their recommendations, and no country simultaneously fulfilled all recommendations. For FBDGs to have a greater impact on diets, clear and consistent policy support is required. Policy measures that could incentivise a greater uptake of FBDGs include investment in targeted health promotion programmes, adopting public procurement standards that are in line with FBDGs, and making sure policies from other governmental departments and ministries are aligned and do not contradict the recommendations of FBDGs—for example, when it comes to national agricultural strategies, public-private partnerships, and regulation of the food sector. In an additional analysis, we show that the value of just the health benefits from adopting progressive FBDGs could amount to 10-25% of national gross domestic product (appendix SI figure 12). This is only a fraction of the current spending on health promotion programmes in many countries.^80^ Increasing the investment in FBDG related measures to a level that is commensurate with the expected benefits would ensure that FBDGs have a more meaningful impact on population health and environmental sustainability than is currently the situation.

**What is already known on this topic**

National food based dietary guidelines (FBDGs) vary in how recommendations are quantified and do not include aspects of sustainability
Adoption of FBDGs could affect health and environmental outcomes, but the environmental evidence is limited to national level analyses covering less than half of all countries with FBDGs, and the health evidence is limited to comparisons with global recommendations

**What this study adds**

Our study suggests that dietary changes towards national FBDGs could be associated with reductions in premature mortality from diet related non-communicable diseases, but the potential benefits could be further improved for most FBDGs
Whereas some FBDGs were associated with reductions in environmental impacts at a national level, the changes were generally moderate
Most FBDGs were not compatible with a set of global environmental targets related to climate change and environmental resource use

## Web extra

Extra material supplied by authors

Supplementary information: appendices S1.1-1.9

Supplementary information: Excel spreadsheet datafile

Supplementary information: Excel spreadsheet with overview of country level results

## Notes

Contributors: MS planned the study, conducted the health and environmental analysis, and wrote the first draft of the manuscript. He acts as the guarantor. MS, LS, and MC reviewed and coded the dietary recommendations. MS and JP prepared the baseline data. All authors analysed and interpreted the results and provided comments on the manuscript. The corresponding author attests that all listed authors meet authorship criteria and that no others meeting the criteria have been omitted.

Ethical approval: Not required. This study is based on population level data that cannot be attributed to individuals. No patients or members of the public were involved in the study.

Data sharing: The country level results generated for the study are available from the Oxford University Research Archive at https://doi.org/10.5287/bodleian:mz8MpewdD. Additional results are available from the corresponding author at marco.springmann@ndph.ox.ac.uk.

The manuscript’s guarantor (MS) affirms that the manuscript is an honest, accurate, and transparent account of the study being reported; that no important aspects of the study have been omitted; and that any discrepancies from the study as originally planned (and, if relevant, registered) have been explained.

Dissemination to participants and related patient and public communities: The results of the study are accessible to the public and will be disseminated and shared with interested parties.

Publisher’s note: Published maps are provided without any warranty of any kind, either express or implied. BMJ remains neutral with regard to jurisdictional claims in published maps.
