# Supplementary material for: The healthiness and sustainability of national and global food based dietary guidelines: modelling study
Source: BMJ. 2020 Jul 15;370:m2322. doi: 10.1136/bmj.m2322 (PMC7362232; doi:10.1136/bmj.m2322)
Supplement: Supplementary file 1 — Supplementary information: appendices S1.1-1.9 [file sprm054404.ww1.pdf]

The healthiness and sustainability of national and global food-based dietary guidelines: modelling study

Supplementary information

Marco Springmann[1], Luke Spajic, Michael A Clark, Joseph Poore, Anna Herforth, Patrick Webb, Mike Rayner, Peter Scarborough

[1]To whom correspondence should be addressed: Address: Nuffield Department of Population Health, University of Oxford, Old Road Campus, Big Data Institute, Headington, Oxford OX3 7LF, UK; Email: marco.springmann@ndph.ox.ac.uk; Phone: +44 (0)7460202512.

Table of Contents

SI.1 Coding scheme ... 2
SI.2 Baseline data ... 7
SI.3 Methods for health analysis ... 12
SI.4 Methods for environmental analysis ... 23
SI.5 Global health and environmental targets ... 25
SI.6 Supplementary results ... 27
SI.7 Uncertainty analysis ... 36
SI.8 Sensitivity analysis ... 38
SI.9 Supplementary economic analysis ... 40
References ... 42

SI.1 Coding scheme

SI Figure 1. Overview of steps followed for analysing the health and environmental impacts of adopting national and global food-based dietary guidelines

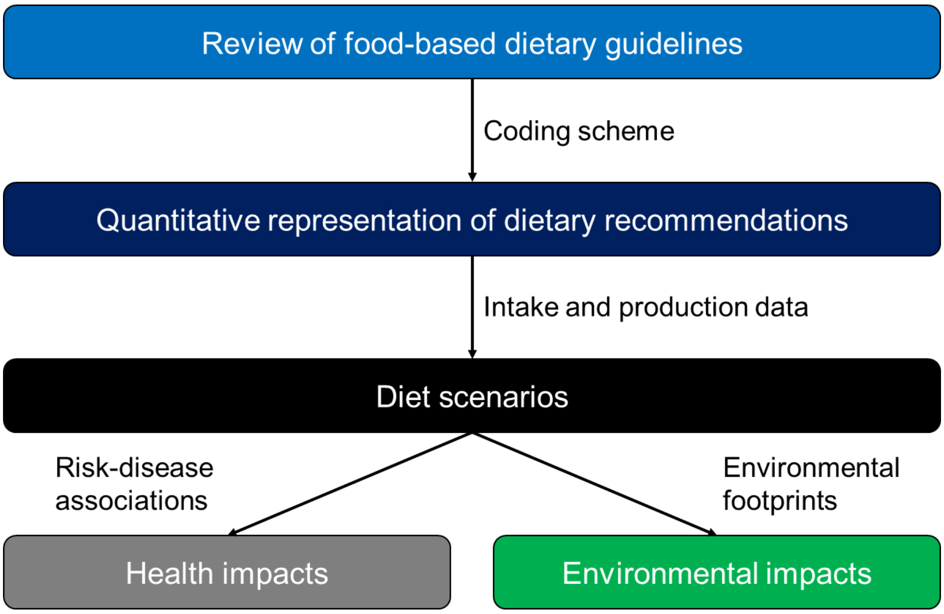

SI Table 1. Countries that submitted FBDGs to FAO repository

Europe	
Belgium-Flanders	Luxembourg
Belgium-Wallonia	Hungary
Bulgaria	Malta
Czechia	Netherlands
Denmark	Austria
Germany	Poland
Estonia	Portugal
Ireland	Romania
Greece	Slovenia
Spain	Slovakia
France	Finland
Croatia	Sweden
Italy	United Kingdom
Cyprus	Switzerland
Latvia	Iceland
Lithuania	Norway
Albania	Israel
Bosnia and Herzegovina	The former Yugoslav Republic of Macedonia
Georgia	Turkey
Asia and Pacific	
Afghanistan	Malaysia
Australia	Mongolia
Bangladesh	Nepal
Cambodia	New Zealand
China	Philippines
Fiji	Republic of Korea
India	Sri Lanka
Indonesia	Thailand
Japan	Viet Nam
Latin America and the Caribbean	
Antigua and Barbuda	El Salvador
Argentina	Grenada
Bahamas	Guatemala
Barbados	Guyana
Belize	Honduras
Bolivia	Jamaica
Brazil	Mexico
Chile	Panama
Colombia	Paraguay
Costa Rica	Saint Kitts and Nevis
Cuba	Saint Lucia
Dominica	Saint Vincent and the Grenadines
Dominican Republic	Uruguay
Ecuador	Venezuela
Africa	
Benin	Seychelles
Kenya	Sierra Leone
Namibia	South Africa
Nigeria	
Near East	
Iran	Oman
Lebanon	Qatar
North America	
Canada	United States

SI Table 2. Coding of uncertainty scores

Uncertainty score	Recommendation	Coded as
1	exact value	exact value
1	range of values	range with mean
1	value with qualifier	value as mean and 20% increase/decrease in high/low values for "at least"/"not more" statements
2	value but serving size is missing	value combined with standard serving size
3	eat daily	if serving size is clear, then code as one serving per day; not coded otherwise
3	value for more general food group	split general recommendation according to regional preference
4	one value across several food groups	assign in proportion to grouping, using serving size for food group of interest
4	eat regularly OR multiple times a week	if serving size is clear (legumes, nuts&seeds, eggs), then range of one serving per week to one serving per day; not coded otherwise
4	increase or decrease intake	increase or decrease by 20% (10-30%), or by value noted
5	vague qualitative recommendation	no change from baseline intake

SI Table 3. Overview of serving sizes by food group

Food group	Serving size (grams)	Comment
Fruits and vegetables	80	Most FBDGs refer to WHO's recommendation of consuming five or more servings of fruits and vegetables (400 g) per day, implicitly assuming a serving size of 80 g. Epidemiological studies likewise use a serving size of 80 g (e.g. Aune et al, 2017).
Total, red and white meat (unprocessed)	30-90	Many FBDGs specified total amounts (e.g. not to consume more than 500 g of red meat per week). When serving sizes were stated, then those differed by region, ranging from 30 g in Asia and the Pacific, over 80 g in Latin America and Africa, to 90 g in Europe. We used those regional serving sizes when recommendations were specified in terms of serving size, but no serving size was provided. FBDGs in North America and the Near East were either fully specified or too unclear to code, so no assumptions on serving sizes were needed.
Legumes (cooked or fresh)	100	A common reference was half a cup of cooked weight (approx 100 g) as serving size. A serving size of 100 g (cooked weight) is in line with assumptions made in epidemiological cohort studies (e.g. Afshin et al, 2014). Whilst stated serving sizes varied across countries, no clear trends between regions were apparent.
Nuts	28	A common reference was a handful of nuts (20-30 g), and there was no clear trend towards other servings sizes across regions. A serving size of 28 g is in line with assumptions made in epidemiological cohort studies (a.g. Aune et al, 2016).
Fish	65-120	Stated serving sizes differed by country and across regions, ranging from 60 g in the Near East and 65 g in Latin America, over 70 g in Asia and the Pacific and 100 g in Africa, to 120 g in Europe. We used those regional values when recommendations were specified in terms of serving sizes, but no serving size was provided.
Milk and yoghurt	130-240	A common reference was one cup, but cup and serving sizes differed across regions, ranging from 130 g in Asia and the Pacific, over 200 g in Latin America and 210 g in Europe and Africa, to 240 g in the Near East and North America. We used those regional serving sizes when recommendations were expressed in terms of serving sizes, but no serving size was provided.
Cheese	30-50	A common reference was a few slices of cheese, but the weight of servings differed across regions, ranging from 30 g in Latin America, over 40 g in Europe and 45 g in the Near East, to 50 g in Africa. We used those regional serving sizes when recommendations were specified in terms of serving sizes, but no serving size was provided.
Egg	50	Most FBDGs used one egg as serving size (approx 50 g). A serving size of 50 g is in line with assumptions made in epidemiological cohort studies (e.g. Zhong et al, 2019).
Whole grains	N/A	Most guidelines did not mentions an exact serving size. We therefore coded recommendations for whole grains as changes in the ratio of whole grains to all grains. The current global average of that ratio is about 15% (Micha et al, 2015).
Processed meat	N/A	Most guidelines did not mention exact serving size. We therefore coded recommendations for processed meat as changes in the ratio between processed meat to the sum of red and processed meat. The current global average of that ratio is about 13% (Micha et al, 2015).

SI Figure 2. Decision tree for including FBDGs in analysis. The FBDGs excluded were Israel, Afghanistan, Nepal, Viet Nam, Antigua and Barbuda, Bahamas, Ecuador, Guyana, Venezuela, Seychelles, Qatar (see SI Datafile). Two subnational FBDGs for Belgium were merged into one national FBDG.

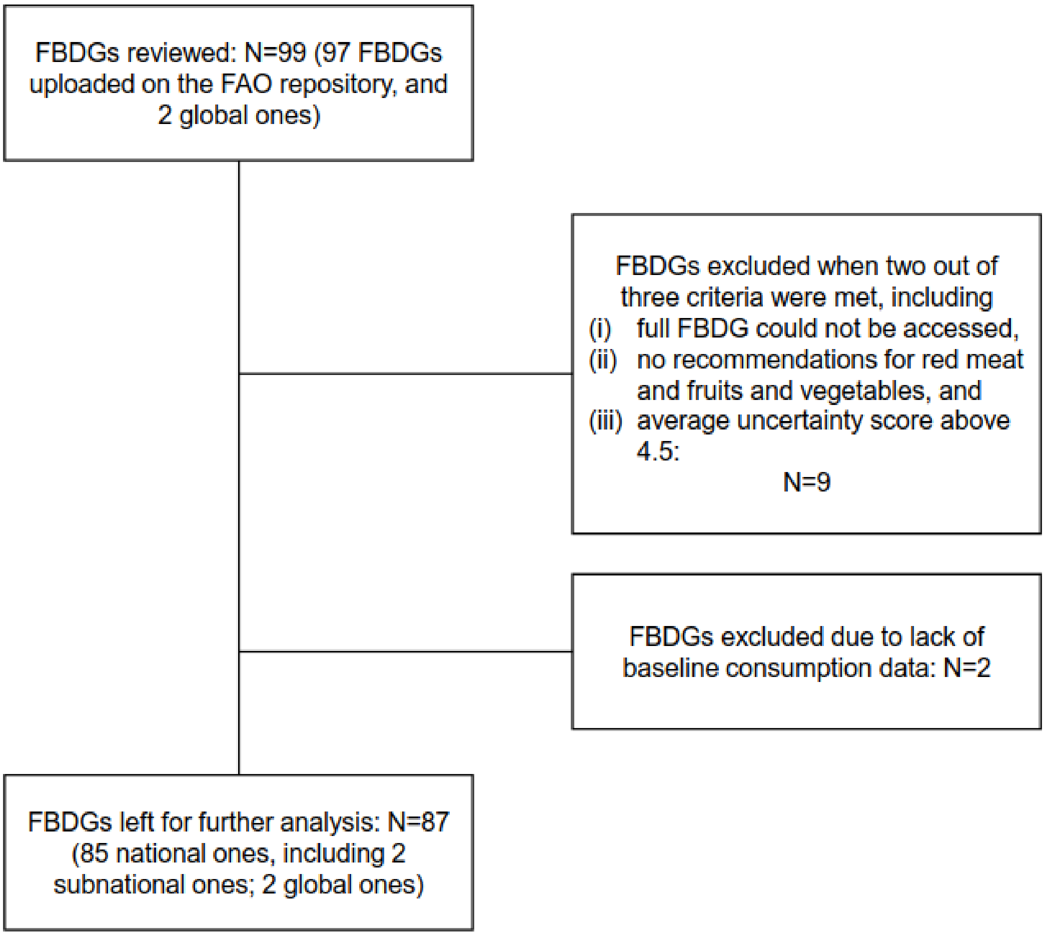

SI.2 Baseline data

SI Table 4. Overview of data inputs and sources.

Type	Coverage	Source	Details
FBDG data:			
FBDG recommendations	Country-level	Source documents of FBDGs accessed via FAO repository (http://www.fao.org/nutrition/education/food-dietary-guidelines/en/).	Main text
Baseline data:			
Food consumption data	Country-level	Food availability data adjusted for food waste at the household level. Estimates of energy intake were in line with trends in body weight across countries.	SI.2
Weight estimates	Country-level	Baseline data from pooled analysis of measurement studies with global coverage. Estimates of optimal energy intake based on age and sex-specific energy needs of a country's population structure.	SI.2
Health analysis:			
Relative risk estimates	General	Adopted from meta-analysis of prospective cohort studies.	SI.3
Mortality and population data	Country-level	Adopted from the Global Burden of Disease project by country and age group.	SI.3
Environmental analysis:			
Environmental footprints	Country-level	Based on global dataset of country and crop-specific environmental footprints for greenhouse gas emissions, cropland use, freshwater ue, and nitrogen and phosphorus application. Footprints for future years account for improvements in technologies, farm-level management, and reductions in food loss and waste.	SI.4
Global health and sustainability targets	Global	Adopted from policy documents and scientific analyses of environmental limits and planetary boundaries.	SI.5

Estimates of optimal energy intake

If FBDGs included recommendations to attain a healthy weight, then this was incorporated by adjusting the intake of staple foods (grains and roots) to attain an energy intake that was in line with optimal BMI levels at a population level. We adopted estimates of optimal energy intake at the population level from Springmann and colleagues.[1] The estimates took into account the age and sex-specific energy needs of each country's population structure, assuming moderate physical activity and the height of the US population as an upper bound.[2,3] We also included the additional energy requirements of pregnancy and lactation,[2] and based the estimates on data on population structure and births from the Global Burden of Disease and the Wittgenstein Centre for Demography and Global Human Capital.[4,5] An overview of optimal energy intake by age group, sex, and region is provided in SI Tables 5-6, and a more detailed description of the methodology is provided by Springmann and colleagues.[1] We note that the exact levels of optimal energy intake used here are of relative minor importance, because the weight adjustment only affects the amount of staple foods in the diet, and staple foods have a low environmental impact when compared to many other foods, and they are also not included as a specific risk factor in our dietary risk assessment.

SI Table 5. Recommended energy intake by age group and sex

Age group	Energy needs (kcal/d)		
	Female	Male	Average
0-4	1200	1200	1200
5-9	1520	1600	1560
10-14	1920	2120	2020
15-19	2040	2760	2400
20-24	2200	2800	2500
25-29	2000	2600	2300
30-34	2000	2600	2300
35-39	2000	2600	2300
40-44	2000	2600	2300
45-49	2000	2400	2200
50-54	1800	2400	2100
55-59	1800	2400	2100
60-64	1800	2400	2000
65-69	1800	2200	2000
70-74	1800	2200	2000
75-79	1800	2200	2000
80-84	1800	2200	2000
85-89	1800	2200	2000
90-94	1800	2200	2000
95-99	1800	2200	2000
100+	1800	2200	2000

SI Table 6. Optimal energy intake at the population level (averaged across all age groups) by region. The energy needs for pregnancy are included in the energy needs for females.

Region	Energy needs (kcal/d)		
	Female	Male	Total
Average for countries with FBDGs	1880	2320	2100
Europe	1880	2350	2110
Latin America and the Carribean	1880	2300	2090
Asia and Pacific	1880	2320	2110
Africa	1840	2190	2020
Near East	1910	2360	2140
North America	1870	2330	2100

Baseline consumption data

We estimated baseline food consumption by adopting estimates of food availability from the FAO's food balance sheets, and adjusting those for the amount of food wasted at the point of consumption.[6,7] We chose food availability data around the year 2010 for our calculation to be consistent with the data on food waste, and the data on the environmental impacts of food production, and we formed a three-year average to reduce the impact of potential misreporting or outlier events in any one year.

Food balance sheets report on the amount of food that is available for human consumption.[7] They reflect the quantities reaching the consumer, but do not include waste from both edible and inedible parts of the food commodity occurring in the household. As such, the amount of food actually consumed may be lower than the quantity shown in the food balance sheet depending on the degree of losses of edible food in the household, e.g. during storage, in preparation and cooking, as plate-waste, or quantities fed to domestic animals and pets, or thrown away.

We followed the waste-accounting methodology developed by the FAO to account for the amount of food wasted at the household level that was not accounted for in food availability estimates.[6] For each commodity and region, we estimated food consumption by multiplying food availability data with conversion factors (*cf*) that represent the amount of edible food (e.g. after peeling) and with the percentage of food wasted during consumption (*1-wp(cns)*). For roots and tubers, fruits and vegetables, and fish and seafood, we also accounted for the differences in wastage between the proportion that is utilised fresh (pct_{frsh}) and the proportion that utilised in processed form (pct_{prcd}). The equation used for each food commodity and region was:

$$Consumption = Availability \cdot \frac{pct_{frsh}}{100} \cdot cf_{frsh} \cdot \left(1 - \frac{wp(cns_{frsh})}{100}\right) + Availability \cdot \frac{pct_{prcd}}{100} \cdot cf_{prcd} \cdot \left(1 - \frac{wp(cns_{prcd})}{100}\right)$$

SI Table 7 provides and overview of the parameters used in the calculation, and SI Table 8 provides an overview of the baseline consumption data calculated in that way. The differences across energy intake reflect differences in the prevalence of overweight and obesity across regions.[8]

Food balance sheets denote food availability in terms of primary commodity equivalents, and therefore do not include estimates of processed foods such as whole grains and processed meat. To be able to code recommendations on whole grains and processed meat, we supplemented our consumption estimates based on waste-adjusted food availability data by estimates from a regionally adjusted set of dietary surveys.[9] For processed meat, we used the survey estimates for red and processed meat to estimate the ratio of processed meat to the sum of red and processed meat, and applied that ratio to our estimates of total red meat intake. For whole grains, no equivalent comparison was available, so we adjusted the estimates for differences in energy intake between the survey results and our estimates, and divided by our estimates of total grain intake to obtain the ratio of whole grain intake.

SI Table 7. Percentage of food wasted during consumption (cns), and percentage of processed utilisation (pct_{prcd}). The percentage of fresh utilisation is calculated as 1-pct_{prcd}. Conversion factors to edible portions of foods are provided below the table.

Food group	Item	Region						
		Europe	USA, Canada, Oceania	Indus-trialized Asia	Sub-Saharan Africa	North Africa, West and Central Asia	South and Southeast Asia	Latin America
cereals	wp(cns)	25	27	20	1	12	3	10
roots and tuber	pct_{prcd}	73	73	15	50	19	10	80
	wp(cns)	17	30	10	2	6	3	4
	wp(cns_{prcd})	12	12	12	1	3	5	2
oilseeds and pulses	cns	4	4	4	1	2	1	2
fruits and vegetables	pct_{prcd}	60	60	4	1	50	5	50
	wp(cns)	19	28	15	5	12	7	10
	wp(cns_{prcd})	15	10	8	1	1	1	1
milk and dairy	wp(cns)	7	15	5	0.1	2	1	4
eggs	wp(cns)	8	15	5	1	12	2	4
meat	wp(cns)	11	11	8	2	8	4	6
fish and seafood	pct_{prcd}	*40% for low-income countries, and 96% for all others.*						
	wp(cns)	11	33	8	2	4	2	4
	wp(cns_{prcd})	10	10	7	1	2	1	2

Conversion factors: maize, millet, sorghum: 0.69; wheat, rye, other grains: 0.78; rice: 1; roots: 0.74 (0.9 for industrial processing); nuts and seeds: 0.79; oils: 1; vegetables: 0.8 (0.75 for industrial processing); fruits: 0.8 (0.75 for industrial processing); beef: 0.715; lamb: 0.71; pork: 0.68; poultry: 0.71; other meat: 0.7; milk and dairy: 1; fish and seafood: 0.5; other crops: 0.78

SI Table 8. Overview of baseline consumption data by region and food group (in grams per day for each food group, and in kcal per day for total energy intake). The regions include all countries with FBDGs (all-NDG), as well as countries with FBDGs in Europe (EURO), Latin America and the Caribbean (LACA), Asia and Pacific (ASPA), Africa (AFRI), the Near East (NEEA), and North America (NOAM). The estimates of grains (wheat, maize, rice, other grains) and red meat (beef, lamb, pork) do not differentiate by the degree of processing and therefore implicitly include whole grains and processed meat. Explicit estimates of the latter are also listed separately.

Food group	Region						
	all-NDG	EURO	LACA	ASPA	AFRI	NEEA	NOAM
wheat	112	182	97	100	66	280	126
rice	149	11	56	209	68	64	14
maize	26	13	83	15	93	4	18
other grains	14	14	5	11	73	2	9
roots	116	140	114	91	380	96	115
legumes	17	10	31	16	26	24	12
soybeans	5	1	4	6	4	0	0
nuts and seeds	9	13	3	8	15	26	17
vegetables	281	231	111	331	118	442	207
fruits (temperate)	77	109	73	73	35	166	87
fruits (tropical)	51	64	110	36	46	101	80
fruits (starchy)	28	14	53	27	34	20	20
vegetable oil	25	45	30	16	13	32	77
palm oil	7	7	7	7	18	1	1
sugar	46	61	90	33	34	57	94
other crops	87	182	125	55	123	18	176
milk	229	581	328	131	88	161	577
eggs	26	29	29	26	10	15	32
beef	19	28	55	8	14	14	66
lamb	5	9	3	4	8	8	2
pork	33	60	22	31	4	0	46
poultry	28	37	60	16	16	48	87
shellfish	8	7	2	9	0	1	12
fish (freshwater)	10	4	3	13	4	5	5
fish (pelagic)	5	6	4	5	6	5	4
fish (demersal)	5	8	3	4	7	2	5
processed meat	13	31	23	6	4	2	49
whole grains	43	56	24	39	63	35	72
energy intake	2245	2428	2335	2174	2134	2334	2563

SI.3 Methods for health analysis

We estimated the mortality and disease burden attributable to dietary and weight-related risk factors by calculating population impact fractions (PIFs) which represent the proportions of disease cases that would be avoided when the risk exposure was changed from a baseline situation to a counterfactual situation. For calculating PIFs, we used the general formula[10–12]:

$$PIF = \frac{\int RR(x)P(x)dx - \int RR(x)P'(x)dx}{\int RR(x)P(x)dx}$$

where $RR(x)$ is the relative risk of disease for risk factor level x, $P(x)$ is the number of people in the population with risk factor level x in the baseline scenario, and $P'(x)$ is the number of people in the population with risk factor level x in the counterfactual scenario. We assumed that changes in relative risks follow a dose-response relationship,[11] and that PIFs combine multiplicatively, i.e. $PIF = 1 - \prod_i (1 - PIF_i)$ where the *i*'s denote independent risk factors.[11,13]

The number of avoided deaths due to the change in risk exposure of risk *i*, $\Delta deaths_i$, was calculated by multiplying the associated PIF by disease-specific death rates, *DR*, and by the number of people alive within a population, *P*:

$$\Delta deaths_i(r,a,d) = PIF_i(r,d) \cdot DR(r,a,d) \cdot P(r,a)$$

where PIFs are differentiated by region *r* and disease/cause of death *d*; the death rates are differentiated by region, age group *a*, and disease; the population groups are differentiated by region and age group; and the change in the number of deaths is differentiated by region, age group and disease.

We used publicly available data sources to parameterize the comparative risk analysis. Mortality data were adopted from the Global Burden of Disease project,[14] and projected forward by using data from the UN Population Division.[15] Baseline data on the weight distribution in each country were adopted from a pooled analysis of population-based measurements undertaken by the NCD Risk Factor Collaboration.[8]

The relative risk estimates that relate the risk factors to the disease endpoints were adopted from meta-analyses of prospective cohort studies for dietary weight-related risks.[16–24] In line with the meta-analyses, we included non-linear dose-response relationships for fruits and vegetables, nuts and seeds, and fish, and assumed linear dose-response relationships for the remaining risk factors. As our analysis was primarily focused on mortality from chronic diseases, we focused on adults aged 20 year or older, and we adjusted the relative-risk estimates for attenuation with age based on a pooled analysis of cohort studies focussed on metabolic risk factors,[25] in line with other assessments.[12,26]

SI Table 9. Relative risk parameters (mean and low and high values of 95% confidence intervals) for dietary risks and weight-related risks. We used non-linear dose-response relationships for fruits and vegetables, nuts and seeds, and fish as specified in the references, and we used linear dose-response relationships for the remaining risk factors.

Food group	Endpoint	Unit	RR mean	RR low	RR high	Reference
Processed meat	CHD	50 g/d	1.27	1.09	1.49	Bechthold et al (2019)
	Stroke	50 g/d	1.17	1.02	1.34	Bechthold et al (2019)
	Colorectal cancer	50 g/d	1.17	1.10	1.23	Schwingshackl et al (2018)
	Type 2 diabetes	50 g/d	1.37	1.22	1.55	Schwingshackl et al (2017)
Red meat	CHD	100 g/d	1.15	1.08	1.23	Bechthold et al (2019)
	Stroke	100 g/d	1.12	1.06	1.17	Bechthold et al (2019)
	Colorectal cancer	100 g/d	1.12	1.06	1.19	Schwingshackl et al (2018)
	Type 2 diabetes	100 g/d	1.17	1.08	1.26	Schwingshackl et al (2017)
Fish	CHD	15 g/d	0.94	0.90	0.98	Zheng et al (2012)
Fruits	CHD	100 g/d	0.95	0.92	0.99	Aune et al (2017)
	Stroke	100 g/d	0.77	0.70	0.84	Aune et al (2017)
	Cancer	100 g/d	0.94	0.91	0.97	Aune et al (2017)
Vegetables	CHD	100 g/d	0.84	0.80	0.88	Aune et al (2017)
	Cancer	100 g/d	0.93	0.91	0.95	Aune et al (2017)
Legumes	CHD	57 g/d	0.86	0.78	0.94	Afshin et al (2014)
Nuts	CHD	28 g/d	0.71	0.63	0.80	Aune et al (2016)
Whole grains	CHD	30 g/d	0.87	0.85	0.90	Aune et al (2016b)
	Cancer	30 g/d	0.95	0.93	0.97	Aune et al (2016b)
	Type 2 diabetes	30 g/d	0.65	0.61	0.70	Aune et al (2016b)
Underweight	CHD	15<BMI<18.5	1.17	1.09	1.24	Global BMI Collab (2016)
	Stroke	15<BMI<18.5	1.37	1.23	1.53	Global BMI Collab (2016)
	Cancer	15<BMI<18.5	1.10	1.05	1.16	Global BMI Collab (2016)
	Respiratory disease	15<BMI<18.5	2.73	2.31	3.23	Global BMI Collab (2016)
Overweight	CHD	25<BMI<30	1.34	1.32	1.35	Global BMI Collab (2016)
	Stroke	25<BMI<30	1.11	1.09	1.14	Global BMI Collab (2016)
	Cancer	25<BMI<30	1.10	1.09	1.12	Global BMI Collab (2016)
	Respiratory disease	25<BMI<30	0.90	0.87	0.94	Global BMI Collab (2016)
	Type 2 diabetes	25<BMI<30	1.88	1.56	2.11	Prosp Studies Collab (2009)
Obesity (grade 1)	CHD	30<BMI<35	2.02	1.91	2.13	Global BMI Collab (2016)
	Stroke	30<BMI<35	1.46	1.39	1.54	Global BMI Collab (2016)
	Cancer	30<BMI<35	1.31	1.28	1.34	Global BMI Collab (2016)
	Respiratory disease	30<BMI<35	1.16	1.08	1.24	Global BMI Collab (2016)
	Type 2 diabetes	30<BMI<35	3.53	2.43	4.45	Prosp Studies Collab (2009)
Obesity (grade 2)	CHD	30<BMI<35	2.81	2.63	3.01	Global BMI Collab (2016)
	Stroke	30<BMI<35	2.11	1.93	2.30	Global BMI Collab (2016)
	Cancer	30<BMI<35	1.57	1.50	1.63	Global BMI Collab (2016)
	Respiratory disease	30<BMI<35	1.79	1.60	1.99	Global BMI Collab (2016)
	Type 2 diabetes	30<BMI<35	6.64	3.80	9.39	Prosp Studies Collab (2009)
Obesity (grade 3)	CHD	30<BMI<35	3.81	3.47	4.17	Global BMI Collab (2016)
	Stroke	30<BMI<35	2.33	2.05	2.65	Global BMI Collab (2016)
	Cancer	30<BMI<35	1.96	1.83	2.09	Global BMI Collab (2016)
	Respiratory disease	30<BMI<35	2.85	2.43	3.34	Global BMI Collab (2016)
	Type 2 diabetes	30<BMI<35	12.49	5.92	19.82	Prosp Studies Collab (2009)

SI Table 9 provides an overview of the relative-risk parameters used, and we provide a detailed discussion of the parameter selection in the following section ("Relative risk parameters"). For ensuring that the relative risks are well-defined for the whole range of exposures considered in the diet scenarios, we capped the maximum exposure/potential risk reductions at the maximum values included in the meta-analyses (800 g/d of fruits or vegetables, 28 g/d of nuts, 50 g/d of fish). For whole-grains, we used a maximum exposure of 125 g/d, in line with the TMREL value suggested by the Global Burden of Disease and the Nutrition and Chronic Diseases Expert Group (NutriCoDE),[26] and we left the linear dose-response functions (for legumes, red meat, and processed meat) unconstraint, but checked that the intake values don't exceed the values covered in the meta-analyses.

The selection of risk-disease associations used in the health analysis was supported by available criteria used to judge the certainty of evidence, such as the Bradford-Hill criteria used by the Nutrition and Chronic Diseases Expert Group (NutriCoDE),[26] the World-Cancer-Research-Fund criteria used by the Global Burden of Disease project,[27] as well as NutriGrade (SI Table 10).[28] The quality of evidence in meta-analyses that covered the same risk-disease associations as used here was graded with NutriGrade as moderate or high for all risk-disease pairs included in the analysis.[19–21] In addition, the Nutrition and Chronic Diseases Expert Group graded the evidence for a causal association of ten of the 14 cardiometabolic risk associations included in the analysis as probable or convincing,[26] and the World Cancer Research Fund graded all five of the cancer associations as probable or convincing.[29] The certainty of evidence grading in each case relates to the general relationship between a risk factor and a health outcome, and not to a specific relative-risk value.

We did not include all available risk-disease associations that were graded as having a moderate certainty of evidence and showed statistically significant results in the meta-analyses that included NutriGrade assessments.[19–21] That was because for some associations, such as for milk, more detailed meta-analyses (with more sensitivity analyses) were available that indicated potential confounding with other major dietary risks.[30,31] Such sensitivity analyses were not presented in the meta-analyses that included NutriGrade assessments, but they are important for health assessments that evaluate changes in multiple risk factors.

For the different diet scenarios, we calculated uncertainty intervals associated with changes in mortality based on standard methods of error propagation and the confidence intervals of the relative risk parameters. For the error propagation, we approximated the error distribution of the relative risks by a normal distribution and used that side of deviations from the mean which was largest. This method leads to conservative and potentially larger uncertainty intervals as probabilistic methods, such as Monte Carlo sampling, but it has significant computational advantages, and is justified for the magnitude of errors dealt with here (<50%) (see e.g. IPCC Uncertainty Guidelines).

SI Table 10. Overview of existing ratings on the certainty of evidence for a statistically significant association between a risk factor and a disease endpoint. The ratings include those of the Nutrition and Chronic Diseases Expert Group (NutriCoDE),[26] the World Cancer Research Fund,[29] and NutriGrade.[19–21] The ratings relate to the risk-disease associations in general, and not to the specific relative-risk factor used for those associations in this analysis.

Food group	Endpoint	Association	Certainty of evidence
Fruits	CHD	reduction	NutriCoDE: probable or convincing;<br>NutriGrade: moderate quality of meta-evidence
	Stroke	reduction	NutriCoDE: probable or convincing<br>NutriGrade: moderate quality of meta-evidence
	Cancer	reduction	WCRF: strong evidence (probable) for some cancers<br>NutriGrade: moderate quality of meta-evidence for colorectal cancer
Vegetables	CHD	reduction	NutriCoDE: probable or convincing<br>NutriGrade: moderate quality of meta-evidence
	Cancer	reduction	WCRF: strong evidence (probable) for non-starchy vegetables and some cancers<br>NutriGrade: moderate quality of meta-evidence for colorectal cancer
Legumes	CHD	reduction	NutriCoDE: probable or convincing<br>NutriGrade: moderate quality of meta-evidence
Nuts and seeds	CHD	reduction	NutriCoDE: probable or convincing<br>NutriGrade: moderate quality of meta-evidence
Whole grains	CHD	reduction	NutriCoDE: probable or convincing<br>NutriGrade: moderate quality of meta-evidence
	Cancer	reduction	WCRF: strong evidence (probable) for colorectal cancer<br>NutriGrade: moderate quality of meta-evidence for colorectal cancer
	Type-2 diabetes	reduction	NutriCoDE: probable or convincing<br>NutriGrade: high quality of meta-evidence
Fish	CHD	reduction	NutriCoDE: probable or convincing<br>NutriGrade: moderate quality of meta-evidence
Red meat	CHD	increase	NutriGrade: moderate quality of meta-evidence
	Stroke	increase	NutriGrade: moderate quality of meta-evidence
	Cancer	increase	WCRF: strong evidence (probable) for colorectal cancer<br>NutriGrade: moderate quality of meta-evidence for colorectal cancer
	Type-2 diabetes	increase	NutriCoDE: probable or convincing<br>NutriGrade: high quality of meta-evidence
Processed meat	CHD	increase	NutriCoDE: probable or convincing<br>NutriGrade: moderate quality of meta-evidence
	Stroke	increase	NutriGrade: moderate quality of meta-evidence
	Cancer	increase	WCRF: strong evidence (convincing) for colorectal cancer<br>NutriGrade: moderate quality of meta-evidence for colorectal cancer
	Type-2 diabetes	increase	NutriGrade: high quality of meta-evidence

NutriCoDE: Nutrition and Chronic Diseases Expert Group

NutriGrade: Grading of Recommendations Assessment, Development, and Evaluation (GRADE) tailored to nutrition research

WCRF: World Cancer Research Fund

Relative risk parameters

Dietary risk factors

Dietary risks are the leading risk factors for death globally and in most regions.[11] The Global Burden of Disease Study included 14 different components as dietary risks, such as not eating enough fruit, nuts and seeds, vegetables, and whole grains, and eating too much red and processed meat. Dietary factors have been associated with the development of cardiovascular diseases, diabetes, and various cancers, and total mortality. In this study, we focused on changes in the consumption of red meat, fish, fruits, vegetables, nuts, and legumes, and we analysed changes in the consumption of whole grains, processed meat, and polyunsaturated fatty acids in a sensitivity analysis. The following provides additional detail and context for the selection of relative risk parameters.

Red and processed meat

In meta-analyses, the consumption of processed meat, including processed beef, pork, and poultry, has been associated with increased risk of coronary heart disease,[32] stroke,[32–36] type 2 diabetes,[32,36,37] cardiovascular diseases in general,[38,39] site-specific cancers,[40–43] total cancer,[39] and all-cause mortality.[38,39,44]

The association between unprocessed red meat and disease risk is generally weaker, but statistically significant for several disease endpoints. In meta-analyses, the consumption of red meat, including beef, lamb, and pork, has been associated with increased risk of coronary heart disease,[45] stroke,[33–35] type 2 diabetes,[37] cardiovascular diseases in general,[38] site-specific cancers [40–43,46], and mortality from all causes,[47] including from CVD and cancer in high-consuming populations [44] and in high-quality studies with long follow-up time [38,39].

There are several plausible explanations for the elevated risks in meat consumers, which support the observational evidence [48]. Mediating factors that are associated with adverse health effects include the composition of dietary fatty acids and cholesterol in red and processed meat, haem iron, as well as sodium, nitrates and nitrites, and advanced glycation end products (AGEs) in processed meats.

For red and processed meat, we adopted linear dose-response relationships between increased intake and increased risk for CHD, stroke, type-2 diabetes, and colorectal cancer from meta-analyses of cohort studies by Bechthold and colleagues,[19] and Schwingshackl and colleagues.[20,21]. The summary relative-risk estimates per 50 g/d increase in processed meat was 1.27 (95% CI, 1.09-1.49; n=3) for CHD, 1.17 (95% CI, 1.02-1.34; n=6) for stroke, 1.37 (95% CI, 1.22-1.55; n=14) for type-2 diabetes, and 1.17 (95% CI, 1.10-1.23; n=16) for colorectal cancer. The summary relative-risk estimates per 100 g/d increase in red meat intake was 1.15 (95% CI, 1.08-1.23; n=3) for CHD, 1.12 (95% CI, 1.06-1.17; n=7) for stroke, 1.17 (95% CI, 1.08-1.26; n=14) for type-2 diabetes, and 1.12 (95% CI, 1.06-1.19; n=21) for colorectal cancer.

White meat

The elevated risks for processed meat also apply to processed white meats, such as processed poultry (and fish). However, the disease associations for unprocessed white meats are less clear. When compared to the baseline diet, there does not seem to be a significant increase in disease risk [38], but substituting other sources of protein with white meat could confer health benefits or detriments, depending on the source of protein that is substituted [49–52]. There are no meta-analyses available that focussed on changes in relative risk from changes in protein sources, but several individual cohort studies provide some guidance. Those indicate that the risk for CHD [50], stroke [49], type 2 diabetes [52] and total mortality [51] can, in part, be reduced for replacement of animal proteins, such as red and processed meat, dairy, poultry, and fish by plant-based protein sources, such as nuts, legumes, and whole grains, but uncertainty intervals were large due to low consumption levels of some of foods.

Dairy

Meta-analyses of prospective cohort studies found no evidence for an association between milk and dairy consumption and mortality from all causes, CHD, and stroke [53–55]. A modest inverse association between milk intake and overall CVD risk was reported by Soedama-Muthu and colleagues [55], but that association was not visible in subgroup analyses, and not replicated in later meta-analyses. Instead, several inconsistencies of that earlier analysis, e.g., with respect to study selection have been identified.[54] Some meta-analyses suggested that milk consumption could reduce the risk of colon cancer [31] and type 2 diabetes [30], but the associations became not statistically significant in each case when adjusted for red and processed meat consumption [30,31]. On the other hand, there is evidence that milk consumption might lead to increased risk of prostate cancer [43,56,57] due to an associated between dairy and insulin-like growth factor 1, an anabolic hormone linked to prostate and other cancers.

Several factors complicate the interpretation of meta-analyses of the health associations of dairy consumption. Three general problems for dairy-related meta-analyses are high heterogeneity of results across individual cohort studies [53,58,59], high degree of potential confounding with other food groups, such as fruits and vegetables and red meat [30,31], and potential conflict of interest in several meta-analyses that were conducted by researchers who received funding from the dairy industry [55,59,60].

It should be noted that milk and dairy consumption is recommended by many nutritional guidelines for meeting nutrient requirements, in particular for calcium. However, the evidence base for such recommendations has been questioned [61], and meta-analyses of randomised controlled trials [62] and observational studies [63] of calcium intake and fracture found no evidence that increasing calcium intake from dietary sources prevents fracture (see also [64]). In addition, lactase persistence, i.e., the ability to digest the milk sugar lactose in adult age, is only present in about a quarter of the world's population, in particular in those from Northern European and Mediterranean decent. The majority of the world's population (70-75%) lose the ability to digest lactose after weaning, which can lead to gastrointestinal symptoms, such as flatulence, bloating, cramps, and diarrhoea upon consumption in some individuals [65–67]. Although lactose intolerance can be managed in a way that milk and dairy

products can be consumed in certain quantities [68], the literature reviewed above does not present a strong case for recommending milk and dairy consumption on health grounds.

Seafood

In meta-analyses of prospective cohort studies, low and moderate consumption of fish has been weakly associated with reduced risk of CHD [22,69], stroke [70,71], mortality from all causes [72], and type 2 diabetes which was mediated by location and fish type [73,74]. For most endpoints, risk reduction of mortality reached a lowest point at or below one serving per day (60-80 g/d), and then levelled off (or turned negative) [72].

Several mechanisms have been suggested to explain the moderate health-protective effect of fish consumption. Fish contains omega-3 fatty acids which have been suggested to lower the risk of all-cause mortality and CHD [72]. Multiple mechanisms of omega 3 fatty acids might be involved, including cell growth inhibition and enhanced apoptosis, suppression of neoplastic transformation and antiangiogenicity. In addition, oily fish contains vitamin D which has been suggested to lower the risk of type 2 diabetes.

With regards to the beneficial impacts of omega-3 fatty acids, a pooled analyses of cohort studies [75] confirmed that an increase in the intake of omega-3 fatty acids is associated with reduced risk of mortality from coronary heart disease, and they also showed that plant-derived omega fatty acids have a similar health benefit as fish-derived fatty acids, which indicates that either source is beneficial and can be substituted.

Subgroup and sensitivity analyses conducted in the meta-analyses of fish consumption and disease risk have highlighted additional aspects, in particular cooking methods and substitution effects. In subgroup analyses, several meta-analyses [71–73] found no statistically significant risk reduction with increased fish consumption in Western countries that consume fish predominantly in fried form, compared to significant risk reductions in Asian countries that consume fish boiled or raw. This finding indicates that cooking methods may play a role in risk mediation. In addition, substitution effects can play a role as fish replaces relatively more unhealthy food groups, such as red and processed meat. The sensitivity analysis by Zhao and colleagues [72] indicated that the statistical significant association between fish consumption and reduction in mortality becomes non-significant if studies adjusted for intakes of red meat, and of fruit and vegetables.

For fish, we adopted a non-linear dose-response relationship between increased intake and reduced risk for CHD from a meta-analysis of cohort studies by Zheng and colleagues.[22] The summary relative-risk estimates per 15 g/d increase in fish intake was 0.94 (95% CI, 0.90-0.98; n=17), with no evidence for further reduction beyond an intake of 50 g/d.

Nuts

In meta-analysis of prospective cohort studies, the consumption of nuts has been associated with reduced risk of CHD [17,76–78], type 2 diabetes by reducing body weight [17,76,77], cardiovascular disease in general [17,77–79], cancer [17,79], mortality from respiratory disease, diabetes, and infections [17], and death from all causes [17,77–79], but not from stroke [17,76,78,80,81].

Most of the reduction in risk was observed for an intake of up to six servings (of 28 g) per week (or 15–20 g/d) for most of the outcomes [17].

The suggested mechanism for the risk reduction from nut consumption includes the fat composition of nuts with low proportions of saturated fatty acids, and high proportions of mono-unsaturated and poly-unsaturared fatty acids which have beneficial effects on inflammation, lipid biomarkers, and blood pressure. Nuts are also a good source of biomarkers which are each associated with reductions in CVD risk, such as folate, antioxidant vitamins and compounds, plant sterols, CA, Mg, and K(7).

For nuts, we adopted non-linear dose-response relationships between increased intake and reduced risk for CHD, type-2 diabetes, and cancer from a meta-analysis of 20 cohort studies by Aune and colleagues.[17] The summary relative-risk estimates per 28 grams/day increase in nut intake were 0.71 (95% CI, 0.63-0.80; n=11) for CHD, 0.61 (95% CI, 0.43-0.88; n=4) for type-2 diabetes, and 0.85 (95% CI, 0.76-0.94; n=8) for cancer. Most of the reduction in risk was observed up to an intake of 15-20 g/d.

Legumes

Less meta-analyses have been conducted about the health associations of changes in the consumption of legumes. Legumes are rich in protein, complex carbohydrates, fiber, and various micronutrients, which could lead to positive health impacts. In one meta-analyses, legume consumption was inversely associated with CHD, but not significantly associated with stroke or diabetes [76]. Another meta-analysis found associations between legume consumption and reduced risk of colorectal cancer [82].

For legumes, we adopted a linear dose-response relationship between increased intake and reduced risk for CHD from a meta-analysis of cohort studies by Afshin and colleagues [76]. The summary relative-risk estimate per 4 weekly 100-g servings was 0.86 (95% CI, 0.78-0.94; n=5).

Fruit and vegetables

In meta-analyses, the consumption of fruits and vegetable has been associated with reduced risk of coronary heart disease [18,83–85], stroke [18,85–87], type 2 diabetes in particular for green leafy vegetables [88,89], cardiovascular disease in general [18,90], mortality from all causes [18,91], and modest reductions in total cancer [18] with greater reductions for site-specific cancers [43,92]. Earlier analyses suggested a threshold of five servings per day above which risks are not reduced further [91], but a recent meta-analyses that included a greater number of studies observed reductions in risk for up to ten servings of fruits and vegetables per day (800 g/d) [18].

Suggested mechanisms include the antioxidant properties of fruits and vegetables that neutralize reactive oxygen species and reduce DNA damage, modulation of hormone metabolism, as well as the benefits from fibre intake on cholesterol, blood pressure and inflammation. Benefits have not been reproducible with equivalent amounts of representative vitamin, mineral and fibre supplements [93,94], which suggests that the micronutrients, phytochemicals, and fibre found in fruits and vegetables act synergistically and through

several biological mechanisms to reduce the risk of chronic disease and premature mortality [95,96].

For fruits and vegetable consumption, we adopted non-linear dose-response relationships between increased intake and reduced risk for CHD, stroke, and cancer from a meta-analysis of 95 cohort studies by Aune and colleagues.[18] The summary relative-risk estimates per 200 grams/day were 0.90 (95% CI, 0.86-0.94; n=26) for fruits and CHD, 0.84 (95% CI, 0.79-0.90; n=23) for vegetables and CHD; 0.82 (95% CI, 0.74-0.90; n=19) for fruits and stroke; 0.96 (95% CI, 0.94-0.99; n=25) for fruits and total cancer, 0.96 (95% CI, 0.93-0.99; n=19) for vegetables and total cancer. For fruits and vegetables combined, the lowest risk for total cancer was observed at an intake of 550-600 g/d, and for CHD and stroke, the lowest risk was observed at 800 g/d, which was at the high end of the range of intake across studies.

Root and tubers

Roots and tubers, such as potatoes and cassava, are the energy stores of plants. In health analyses, they are often not classified as vegetables due to their high starch content and comparatively lower content of vitamins, minerals, and phytochemicals [43], and together with starchy fruits, such as bananas and plantains, are considered a separate category. Although roots and tubers do not appear to have similarly beneficial health impacts as non-starchy fruits and vegetables, there is inconsistent evidence from meta-analyses that roots and tubers are detrimental for health per se, or whether it is the added fats in Western-style consumption patterns, such French fries, that contribute to observed negative health impacts [97–99].

Grains

The health impacts of grain consumption depend on the degree of processing. Milling whole grains to refined grains removes the germ and ban from the endosperm. Whole grains, but not refined grains, have been associated in meta-analyses with reduced risk of cardiovascular disease [100,101], coronary heart disease [100,102], cancer [24], type 2 diabetes [100,103], and other causes of death [24]. Their consumption has also been associated with reductions in overweight and obesity [102].

Suggested mechanisms refer to the fibre content of whole grains which reduces glucose and insulin responses, lowers concentration of total and low density lipoprotein (LDL) cholersterol, improves the functional properties of the digestive tract (binding, removing, excretion), and decreases inflammatory markers [24].

The consumption of refined grains has, in most cases, not been consistently associated with disease outcomes in meta-analyses [100,104,105], but replacement of refined grains with whole grains would confer reductions in the risks of cardiovascular disease, cancer, and type 2 diabetes as reviewed above.

For whole grains, we adopted non-linear dose-response relationships between increased intake and reduced risk for CHD, type-2 diabetes, and cancer from a meta-analysis of 45 cohort studies by Aune and colleagues [24]. The summary relative-risk estimates per 30

grams/day increase in whole grain intake were 0.87 (95% CI, 0.85-0.90; n=3) for CHD, 0.65 (95% CI, 0.61-0.70; n=4) for type-2 diabetes, and 0.95 (95% CI, 0.93-0.97; n=6) for cancer.

Oils and fats

In meta-analyses of prospective cohorts, the consumption of trans fats, in particular from hardened vegetable oil, has been clearly associated with increased risk of CHD [106,107], and all-cause mortality [106]. However, the health effects of other oils and fats depend on what they replace in the diet. In meta-analyses of randomised controlled trials and prospective cohorts, replacing saturated fatty acids, which is present in large proportions in butter and dairy fats, by polyunsaturated fatty acids, which is present predominantly in vegetable oils and nuts as omega-6 fatty acids, and seafood and seeds as omega-3 fatty acids, reduced risk of CHD [75,108–110], whereas replacement by refined carbohydrates increased CHD risk in cohort studies [109] but not in RCTs [110], and no consistent association was found for replacement by monounsaturated fatty acids [109,111].

The health impacts are broadly consistent with effects on blood lipids [112,113], and with modelling studies based on those relationships [107]. The greater the ratio of total cholesterol (TC) (which is the sum of low-density lipoprotein, LDL, and high-density lipoprotein, HDL) to HDL cholesterol, the greater the risk of CHD. Substituting saturated fatty acids by refined carbohydrates reduces HDL and therefore increases the TC:HDL ratio, whereas substituting saturated fatty acids by polyunsaturated fatty acids reduces LDL and therefore reduced the TC:HDL ratio. Trans fat both increases LDL and decreases HDL, and therefore leads to greater increases in the TC:HDL ratio and risk of CHD than other fats.

The degree to which substitution between different food sources of fatty acids contribute to CHD risk has received less attention, despite the fact that foods generally contain a mix of different fatty acids. Chen and colleagues assessed the health effects of replacing dairy fat from milk, ice cream, yoghurt, cheese, and cream [114]. In their analysis of three US cohorts, they found greatest reductions of CVD risk (including CHD and stroke) for replacement of dairy fat by carbohydrates from whole grains, followed by vegetables fats, neutral effects for replacement by refined starches, and increased risk for replacement by other animal fats, such as lard. When polyunsaturated fatty acids were analysed separately, the greatest risk reduction was seen for plant-based omega-6 fatty acids, followed by plant-based omega-3 fatty acid (alpha-linolenic acid), and then marine-based omega-3 fatty acids which was associated with the lowest risk reduction. These results support recommendations to replace animal fats, including dairy fats, with vegetable sources of fats in the prevention of CVD [114].

Sugar

In meta-analyses of prospective cohort studies and randomised controlled trials, the consumption of free (added) sugars and sugar sweetened beverages has been associated with weight gain [115,116] and metabolic syndrome, a cluster of cardio-metabolic risk factors that are predictive of CVD [117,118]. In meta-analyses of prospective cohort studies, sugar sweetened beverages in particular were also associated with increased risk of type 2 diabetes independent of weight gain [119]. Increased risk of type 2 diabetes was also observed for artificially sweetened beverages and fruit juice, but study quality was judged to be low in each case [119].

The underlying mechanisms that have been suggested include incomplete compensation for liquid calories from sugar sweetened beverages, and a high glycemic load from free sugars, both of which lead to weight gain [115,120]. Increased diabetes and cardiovascular disease risk also occur independently of weight through adverse glycemic effects and increased fructose metabolism in the liver [120].

Weight-related risk factors

Excess weight is an established risk factor for several causes of death, including ischaemic heart disease,[121,122] stroke,[122–124] and various cancers.[43,125–127] Plausible biological explanations[128–130] and the identification of mediating factors[130,131] suggest that the association between body weight and mortality is not merely statistical association, but a causal link independent of other factors, such as diet and exercise.[132–136]

We inferred the parameters describing relative mortality risk due to weight categories from two large, pooled analyses of prospective cohort studies.[130,137] We adopted the relative risks for coronary heart disease, stroke, cancer, and respiratory disease from the Global BMI Mortality Collaboration, which conducted a participant-data meta-analysis of 239 prospective studies in four continents,[23] and we adopted the relative risks for type-2 diabetes from the Prospective Studies Collaboration,[130] which analysed the association between BMI and mortality among 900,000 persons in 57 prospective studies. From each study, we adopted the relative risk rates for lifelong non-smokers and excluding the first 5 years of follow-up to minimize confounding and reverse causality. Although most data used in those meta-analyses stemmed from Western cohorts, the risk-disease association were broadly similar in different populations wherever overweight and obesity were common.[23]

SI.4 Methods for environmental analysis

We estimated the environmental impacts of adopting FBDGs by using a global dataset of country and crop-specific environmental footprints for greenhouse gas (GHG) emissions, cropland use, freshwater use, and nitrogen and phosphorus application.[138] The footprints are based on global datasets on environmental resource use in the producing region,[139–142] which have been converted to consumption-related footprints by using a food systems model that connects food production and consumption across regions.[138] The model distinguished several steps along the food chain: primary production, trade in primary commodities, processing to oils, oil cakes and refined sugar, use of feed for animals, and trade in processed commodities and animals. It was parameterised with data from the International Model for Policy Analysis of Agricultural Commodities and Trade (IMPACT) [139] on current and future food production, processing factors, and feed requirements for 62 agricultural commodities and 159 countries. Projections of future food consumption and production were based on statistical association with changes in income and population, and were in line with other projections.[143] A full description of the model and the IMPACT-related parameters is provided elsewhere.[138,139] SI Table 11 provides an overview of the footprints, and we provide short descriptions for each environmental domain below.

For GHG emissions, we focused on the non-CO_2 emissions of agriculture, in particular methane and nitrous oxide, in line with methodology followed by the International Panel on Climate Change. Data on GHG emissions were adopted from country-specific analyses of GHG emissions from crops,[141] and livestock.[144] Non-CO_2 emissions of fish and seafood were calculated based on feed requirements and feed-related emissions of aquaculture,[145] and on projections of the ratio between wild-caught and farmed fish production.[146,147] For future years, we incorporated the mitigation potential of bottom-up changes in management practices and technologies by using marginal abatement cost curves,[148] and the projected value of the social cost of carbon (SCC) in that year.[149] The mitigation options included changes in irrigation, cropping and fertilization that reduce methane and nitrous oxide emissions for rice and other crops, as well as changes in manure management, feed conversion and feed additives that reduce enteric fermentation in livestock.

Data on cropland and consumptive freshwater use from surface and groundwater (also termed blue water) were adopted from the IMPACT model for a range of different socio-economic pathways.[139] To derive commodity-specific footprints, we divided use data by data on primary production, and we calculated the footprints of processed goods (vegetable oils, refined sugar) by using country-specific conversion ratios,[139] and splitting coproducts (oils and oil meals) by economic value to avoid double counting. We used country-specific feed requirements for terrestrial animals [139] to derive the cropland and freshwater footprints for meat and dairy, and we used global feed requirements for aquaculture [145] and projections of the ratio between wild-caught and farmed fish production [146,147] to derive the cropland and freshwater footprints for fish and seafood. For future years, we included efficiency gains in agricultural yields, water management, and feed conversion that were based on IMPACT projections.[139] For water management, we relied on an integrated hydrological model within IMPACT that operates at the level of watersheds and accounts for management changes that increase basin efficiency, storage capacity, and better utilization of rainwater.[139] For agricultural yields, the gains in land-use efficiency by 2050 matched estimates of yield-gap

closures of about 75% between current yields and yields that are feasible in a given agro-climatic zone.[150]

SI Table 11. Environmental footprints of food commodities (per kg of product) (global averages) for the years 2010 and 2050. Footprints for animal products represent feed-related impacts, except for GHG emissions of livestock which also have a direct component. Footprints for fish and seafood represent feed-related impacts of aquaculture production weighted by total production volumes. The global averages account for expected efficiency improvements, such as improved feed for livestock, and changes in production by 2050, such as increases in extensive beef production in middle-income countries. The analysis is based on country-specific values.

Food group	GHG emissions ($kgCO_2eq/kg$)		Cropland use (m^2/kg)		Freshwater use (m^3/kg)		Nitrogen use (kgN/t)		Phosphorus use (kgP/t)	
	2010	2050	2010	2050	2010	2050	2010	2050	2010	2050
wheat	0.23	0.21	3.36	2.46	0.49	0.37	28.73	19.78	4.39	2.01
rice	1.18	0.90	3.51	2.78	1.07	0.89	36.64	25.07	5.20	2.28
maize	0.19	0.17	1.98	1.40	0.15	0.12	22.77	14.36	3.57	1.55
other grains	0.29	0.22	6.17	4.43	0.17	0.14	16.39	9.82	2.72	0.97
roots	0.07	0.06	0.69	0.52	0.04	0.04	3.60	2.07	0.71	0.30
legumes	0.23	0.19	11.11	6.89	0.94	0.61	0.00	0.00	0.00	0.00
soybeans	0.12	0.09	3.95	3.14	0.14	0.15	2.75	1.75	5.88	3.17
nuts&seeds	0.69	0.65	6.39	5.13	0.43	0.33	14.16	10.84	2.10	1.17
vegetables	0.06	0.07	0.49	0.34	0.09	0.06	9.55	6.32	1.67	0.81
oilcrops	0.70	0.64	3.12	2.37	0.22	0.19	13.33	8.50	2.86	1.32
fruits (temperate)	0.08	0.08	1.18	0.97	0.33	0.28	12.73	8.57	1.91	0.92
fruits (tropical)	0.09	0.10	0.94	0.62	0.32	0.23	10.27	6.10	1.58	0.70
fruits (starchy)	0.11	0.10	0.88	0.59	0.11	0.08	6.15	3.76	1.05	0.48
sugar	0.19	0.19	1.67	1.35	1.22	0.88	22.34	15.26	3.84	1.86
palm oil	1.85	2.03	3.10	2.39	0.00	0.00	22.34	16.29	3.57	1.85
vegetable oil	0.67	0.63	10.31	8.46	0.47	0.45	42.73	28.19	11.47	5.66
beef	36.78	40.36	4.21	2.78	0.22	0.17	27.29	17.16	5.36	2.29
lamb	36.73	37.21	6.24	4.48	0.49	0.42	27.52	21.82	4.94	2.47
pork	3.14	3.25	6.08	4.90	0.35	0.29	51.52	34.19	8.87	4.05
poultry	1.45	1.39	6.59	5.18	0.40	0.36	50.20	36.00	9.02	4.35
eggs	1.61	1.48	6.86	5.19	0.44	0.39	51.22	35.09	8.81	4.18
milk	1.28	1.39	1.34	1.01	0.08	0.08	6.32	4.63	1.58	0.78
shellfish	0.03	0.04	0.36	0.46	0.03	0.04	2.19	2.39	0.50	0.40
fish (freshwater)	0.12	0.12	1.51	1.37	0.10	0.10	11.26	8.39	2.37	1.29
fish (pelagic)	0.00	0.00	0.00	0.00	0.00	0.00	0.00	0.00	0.00	0.00
fish (demersal)	0.01	0.01	0.13	0.20	0.01	0.01	0.75	0.99	0.19	0.18

Data on fertilizer application rates of nitrogen and phosphorous were adopted from the International Fertilizer Industry Association [142]. For future years, we included efficiency gains in nitrogen and phosphorus application from rebalancing of fertilizer application rates between over and under-applying regions in line with closing yield gaps.[150] In addition, we

included improvements in nitrogen use efficiency of 15% by 2030 and 30% by 2050, in line with targets suggested by the Global Nitrogen Assessment,[151] and we included recycling rates of phosphorus of 25% by 2030 and 50% by 2050.[152]

SI.5 Global health and environmental targets

We analysed whether the FBDGs were in line with global health and environmental targets by modelling their universal adoption across all 169 countries that we have consumption and environmental data for. With the exception of the proportional NCD target, all targets were expressed in absolute terms, e.g. not exceeding global GHG emissions (related to food consumption) of a certain amount. In context of these absolute targets, the rationale of the global sustainability test is to assess whether global targets can be met without imposing exceptions for one country or group of countries. From this equity perspective, a country whose FBDG fails the test is, in effect, outsourcing its responsibility towards fulfilling the target, and other countries would have to divert from the FBDG to meet it.

The targets included are the Sustainable Development Goal of reducing premature mortality from non-communicable diseases (NCDs) by a third, the Paris Agreement to limit global warming to below 2 degrees Celsius, the Aichi Biodiversity Target of limiting the rate of land-use change, as well as the Sustainable Development Goals and planetary boundaries related to freshwater use, and nitrogen and phosphorus pollution (SI Table 12).

For deriving the target values, we isolated the diet-related portion of the different health and environmental targets, such as the emissions budget allocated to food production under a climate stabilisation pathway that is in line with fulfilling the Paris Climate Agreement,[153] which mirrored how the planetary boundaries for the food system were derived from the overall boundary values.[138] For NCD risks, we took into account what proportion of NCD risks are due to dietary risks.[154] When targets were expressed for future years, we used projections of environmental footprints that included improvements in technologies and management practices, including reductions in food loss and waste, along a middle-of-the-road socio-economic development pathway.[138] We summarise the derivation of the target values below.

SI Table 12. Overview of global health and environmental targets and their derivation.

Global targets	Comment	Implementation
NCD Agenda	The Sustainable Development Goal (SDG) 3.4 is to "reduce by one third premature mortality from NCDs through prevention and treatment, and promote mental health and wellbeing", which builds on the World Health Organization (WHO) "25x25" NCD target.	According to the Global Burden of Disease project (GBD 2017), imbalanced diets and weight contribute more than half to preventable causes of NCD deaths (the rest is tobacco, alcohol, and physical activity). Applying this proportion to overall reductions yields a target for diet-related reductions of around 18.5%.
Paris Climate Agreement	The Paris Agreement's long-term goal is to keep the increase in global average temperature to well below 2 °C above pre-industrial levels; and to limit the increase to 1.5 °C, since this would substantially reduce the risks and effects of climate change. The goal is reflected in SDG 13 and in the plenatary boundary for climate change.	The target for agricultural emissions in line with the 2 degree target was derived as 4.7 (4.3-5.3) $GtCO_2$-eq (Wollenberg et al, 2016; Springmann et al, 2018). We adjusted this value for the proportion of emissions related specifically to food consumption (92% of emissions of the whole food system, according to Springmann et al, 2018).
Aichi Biodiversity Targets	Target 5: By 2020, the rate of loss of all natural habitats, including forests, is at least halved and where feasible brought close to zero, and degradation and fragmentation is significantly reduced. The target is related to SDG 15 and the planetary boundary for land-system change.	Contribute to target by not increasing pressure to convert natural land into cropland (or pastures), in line with the food-related planetary boundary for land-systems change (Steffen et al, 2015; Springmann et al, 2018). The planetary boundary value was set to the extent of current cropland (+/- 16%). We internally recalculated the value for consistency with the baseline parameters and our focus on food available for consumption (9.9 Mkm^2, 8.3-11.5).
SDG target on water withdrawals	SDG 6.4: By 2030, substantially increase water-use efficiency across all sectors and ensure sustainable withdrawals and supply of freshwater to address water scarcity and substantially reduce the number of people suffering from water scarcity. The goal is in line with the planetary boundary for freshwater use.	Adopt the food-related planetary-boundary target of maintaining environmental flow requirements by limiting agricultural freshwater use to below 2,000 $km^{3,}$ with a range of 800-3350 km^3 (Springmann et al, 2018). We adjusted the value for the proportion of the food system attributed to diets (1,600 km^3, 640-2600).
SDG target on nutrient pollution	SDG 14.1: By 2025, prevent and significantly reduce marine pollution of all kinds, in particular from land-based activities, including marine debris and nutrient pollution. The goal is in line with the planetary boundary for biogeochemical flows of nitrogen and phosphorus.	Adopt the food-related planetary-boundary target for nitrogen and phosphrus application in line with limiting eutrophication risk (de Vries et al, 2013; Springmann et al, 2018). We recalculated the value for our focus on consumption-related impacts by applying the original risk fractions to estimates of baseline use, which yielded target values of 51 TgN (38-83) and 11 TgP (5.6-12.9).

SI.6 Supplementary results

SI Table 13. Overview of coding scores by WHO region and food group. The food groups were coded on a scale of 1 (low uncertainty) to 5 (high uncertainty), whilst recommendations on energy balance were coded on a scale of 1 (recommended) and 0 (not mentioned). Uncertainty scores were averaged across recommendations for fruits and vegetables, legumes, nuts and seeds, whole grains, milk, eggs, fish, sugar, red meat, processed meat.

Food group	Regions of national FBDGs							Global FBDGs	
	Average	Europe	North America	Near East	Asia and Pacific	Latin America	Africa	WHO	EAT-Lancet
Total	3.2	2.9	3.0	3.0	3.3	3.4	3.8	4.0	1.0
Fruits&veg	1.9	1.6	3.0	2.0	1.7	2.1	2.5	1.0	1.0
Milk	2.3	1.6	3.0	2.0	2.3	2.8	3.7	5.0	1.0
Sugar	2.8	2.9	1.0	2.0	2.9	2.7	3.8	1.0	1.0
Fish	2.9	2.1	3.0	2.3	3.4	3.7	3.7	5.0	1.0
Legumes	3.2	3.5	2.5	2.0	3.1	3.0	3.5	5.0	1.0
Eggs	3.3	3.1	3.0	4.3	2.9	3.5	4.2	5.0	1.0
Red meat	3.4	2.9	4.5	4.0	3.8	3.7	3.8	5.0	1.0
Nuts&seeds	3.8	3.2	2.5	4.7	4.4	4.0	4.5	5.0	1.0
Whole grains	3.9	3.7	2.5	3.0	3.9	4.3	4.2	4.0	1.0
Processed meat	4.2	4.6	5.0	3.3	4.5	3.8	3.8	4.0	1.0
Energy balance	0.8	0.7	1.0	1.0	0.9	0.9	0.7	1.0	1.0

SI Figure 3. Number of countries (%) meeting guidelines on recommended and discouraged food groups. The guidelines include national FBDGs, and global ones, including the EAT-Lancet recommendations on healthy and sustainable diets (EAT) and WHO recommendations (WHO). The total number of countries with FBDGs is 85, including 36 from Europe, 23 from Latin America and the Caribbean, 15 from Asia and the Pacific, 6 from Africa, 3 from the Near East, and 2 from North America.

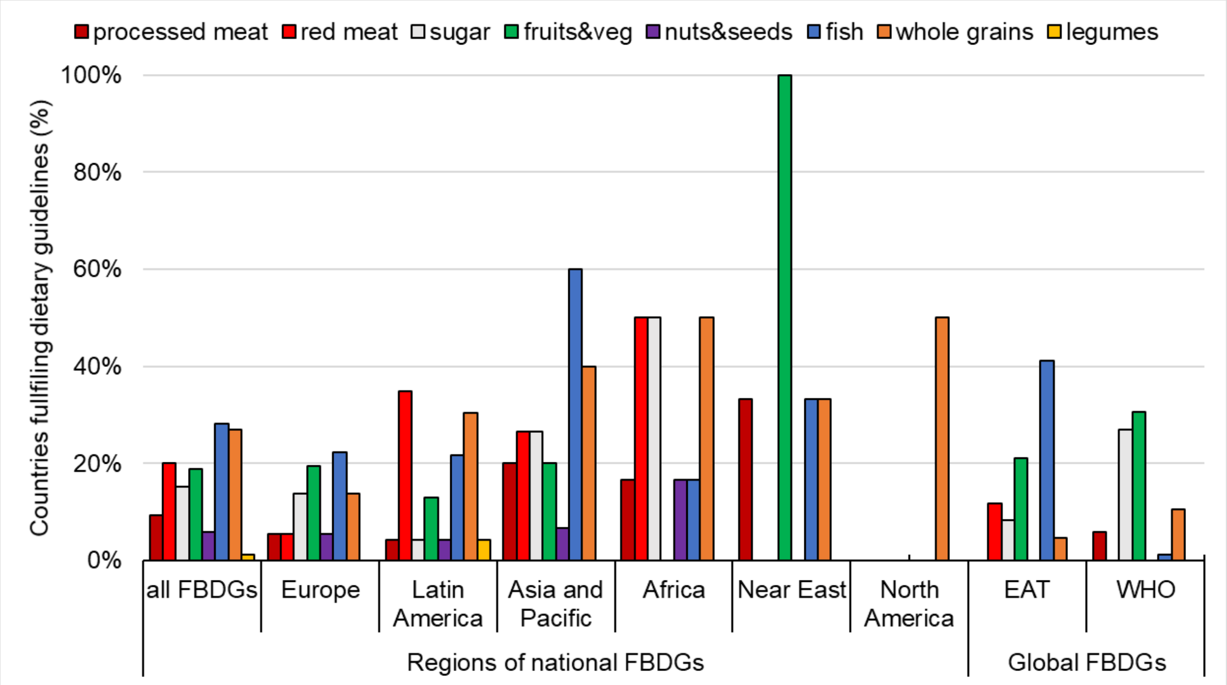

SI Figure 4. Reduction in premature mortality attributed to avoided causes of death (% of total). The causes of death include coronary heart disease (CHD), stroke, cancer, type 2 diabetes (T2DM), and respiratory disease. Absolute numbers are listed in SI Datafile 2.

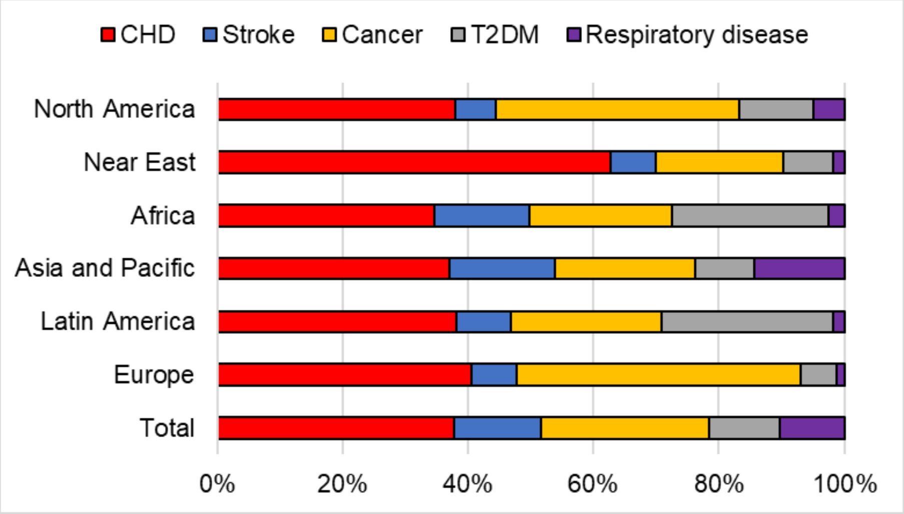

SI Figure 5. Reductions in premature mortality (%) for adopting national FBDGs (upper panel), WHO recommendations (middle panel), and the EAT-Lancet recommendations (lower panel) by country.

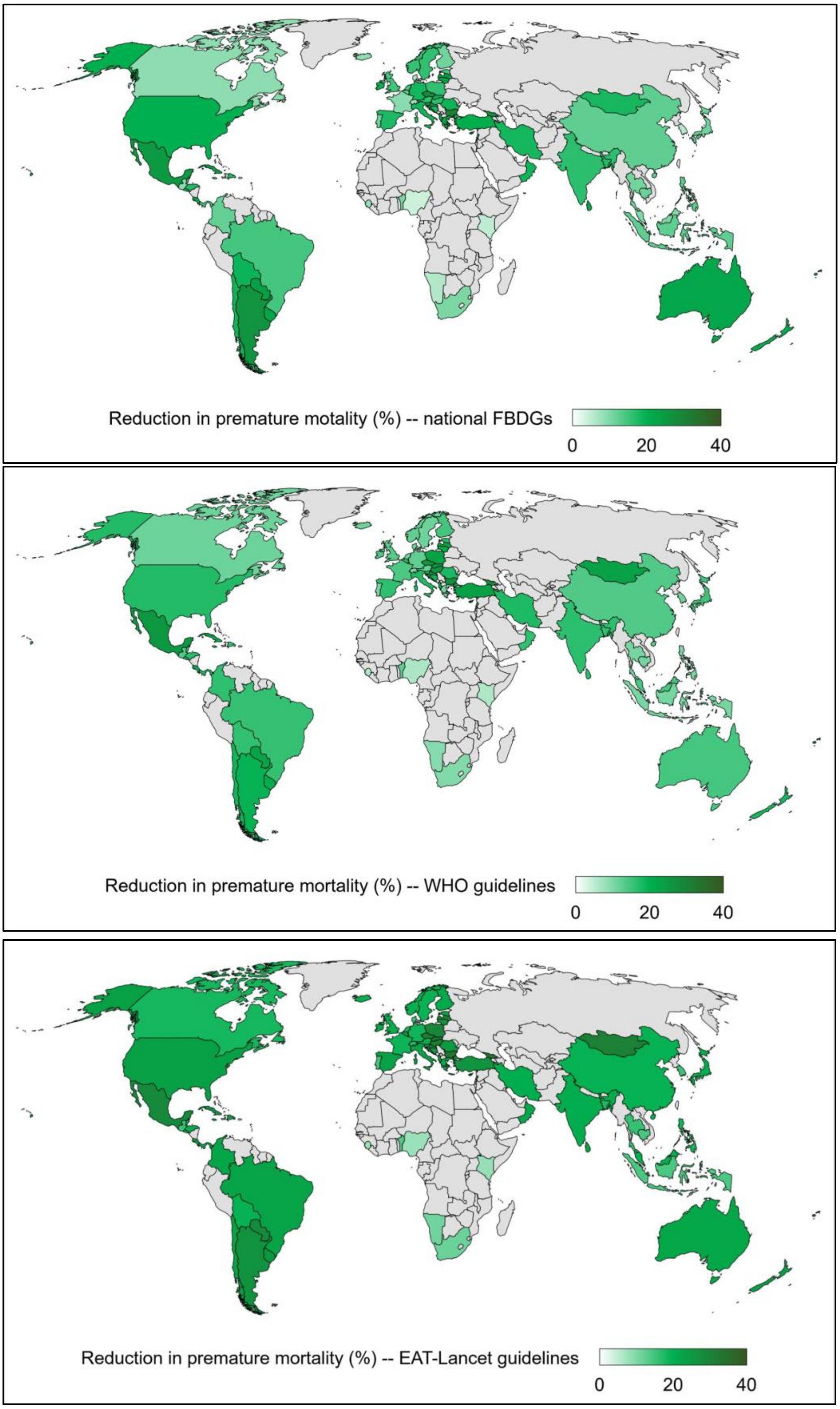

SI Figure 6. Change in environmental resource demand (%) for adopting national FBDGs by country.

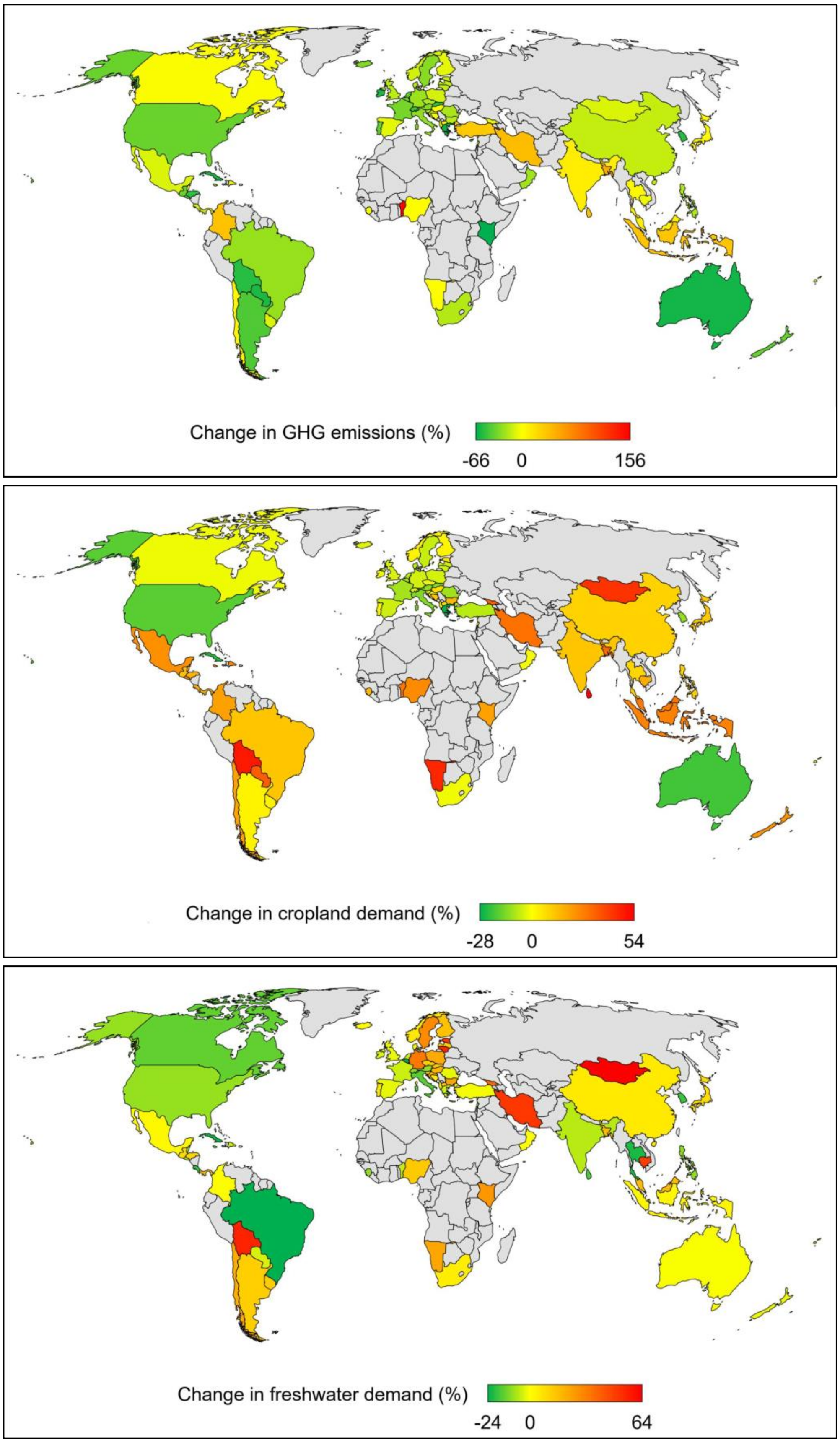

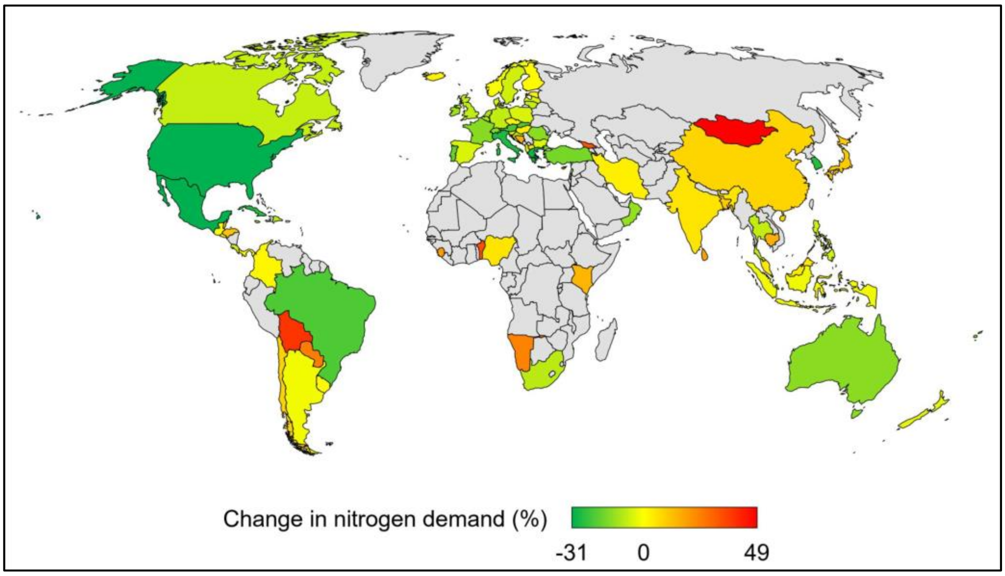

Change in nitrogen demand (%)
-31
0
49

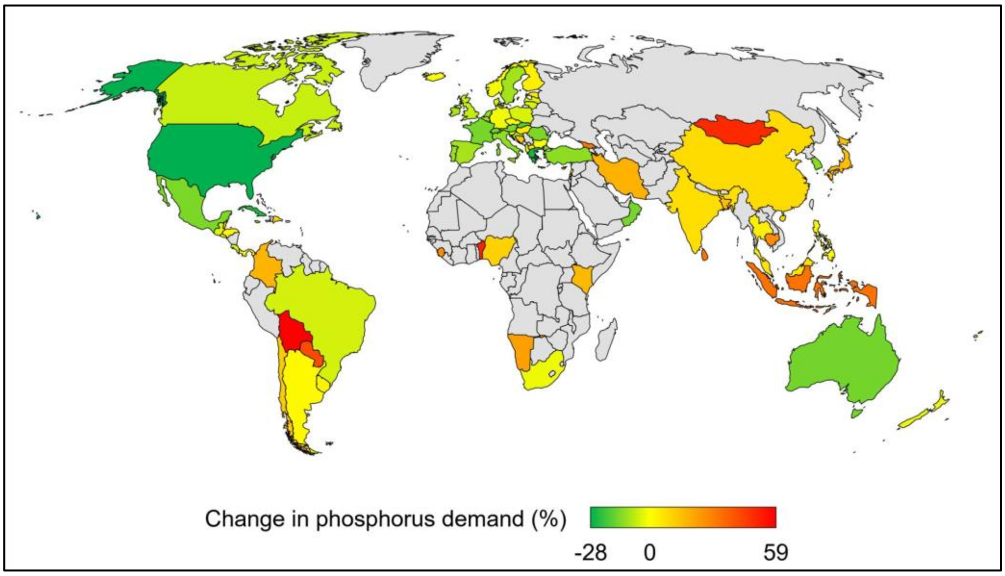

Change in phosphorus demand (%)
-28
0
59

SI Figure 7. Global environmental impacts (as percentage of global environmental target) of adopting FBDGs by FBDGs region, environmental domain, and food group. The global environmental impacts by FBDG regions are calculated by first estimating the global impacts of universal adoption of each country's national FBDG, and then averaging those global impacts across FBDGs in each aggregate region. The impacts of the global FBDGs (WHO, EAT) are calculated by summing the environmental impacts in each country (the impacts of averaging across aggregate region is shown in SI Figure 8).

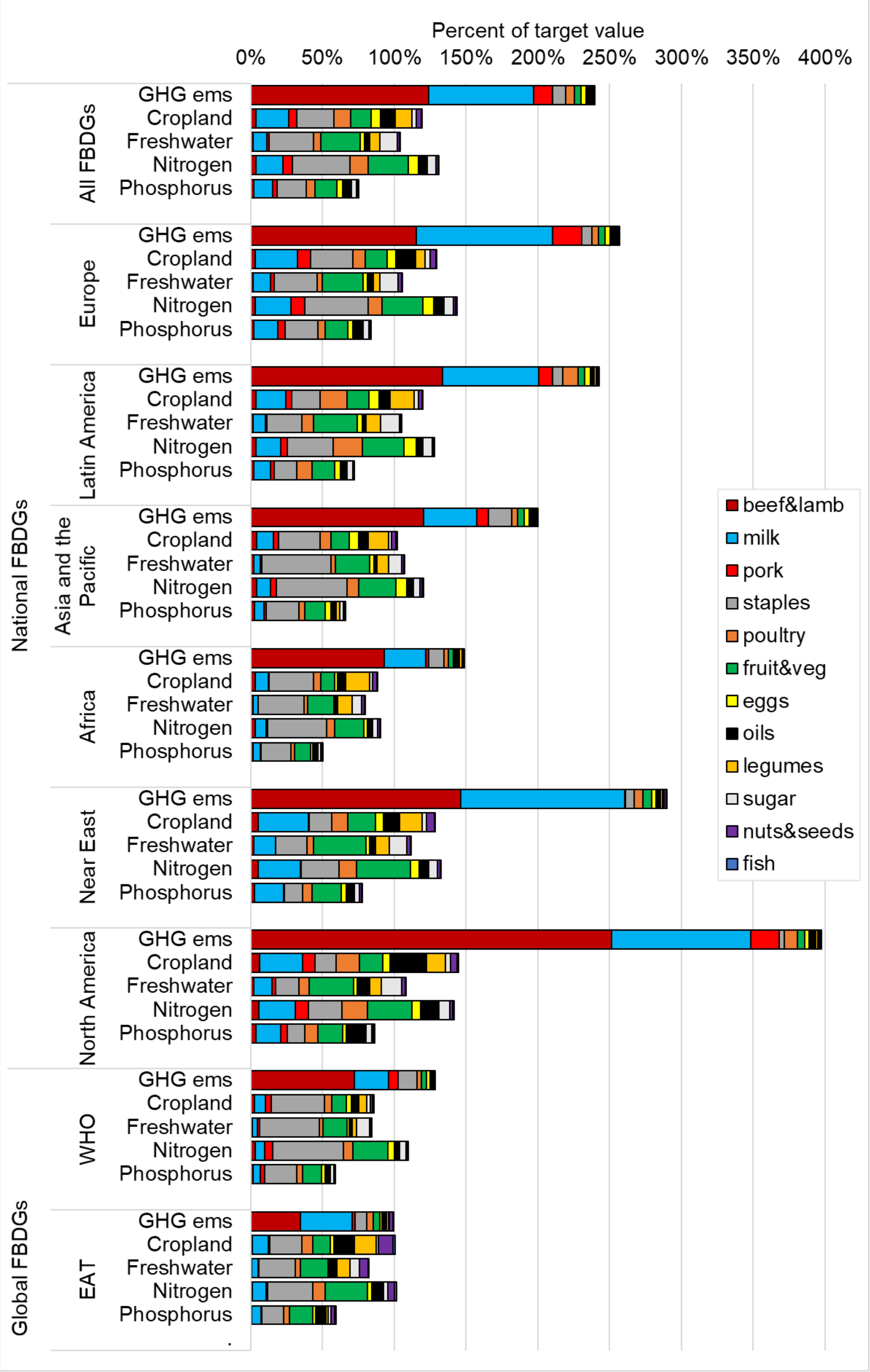

SI Figure 8. Global environmental impacts (as percentage of global environmental target) by FBDGs region, environmental domain, and scenario. The scenarios include baseline diets (BMK), national FBDGs (NDG), and the global recommendations of the WHO and the EAT-Lancet Commission. The global environmental impacts by FBDG regions are calculated by first estimating the global impacts of universal adoption of each country's diet scenario, and then averaging those global impacts across FBDGs in each aggregate region.

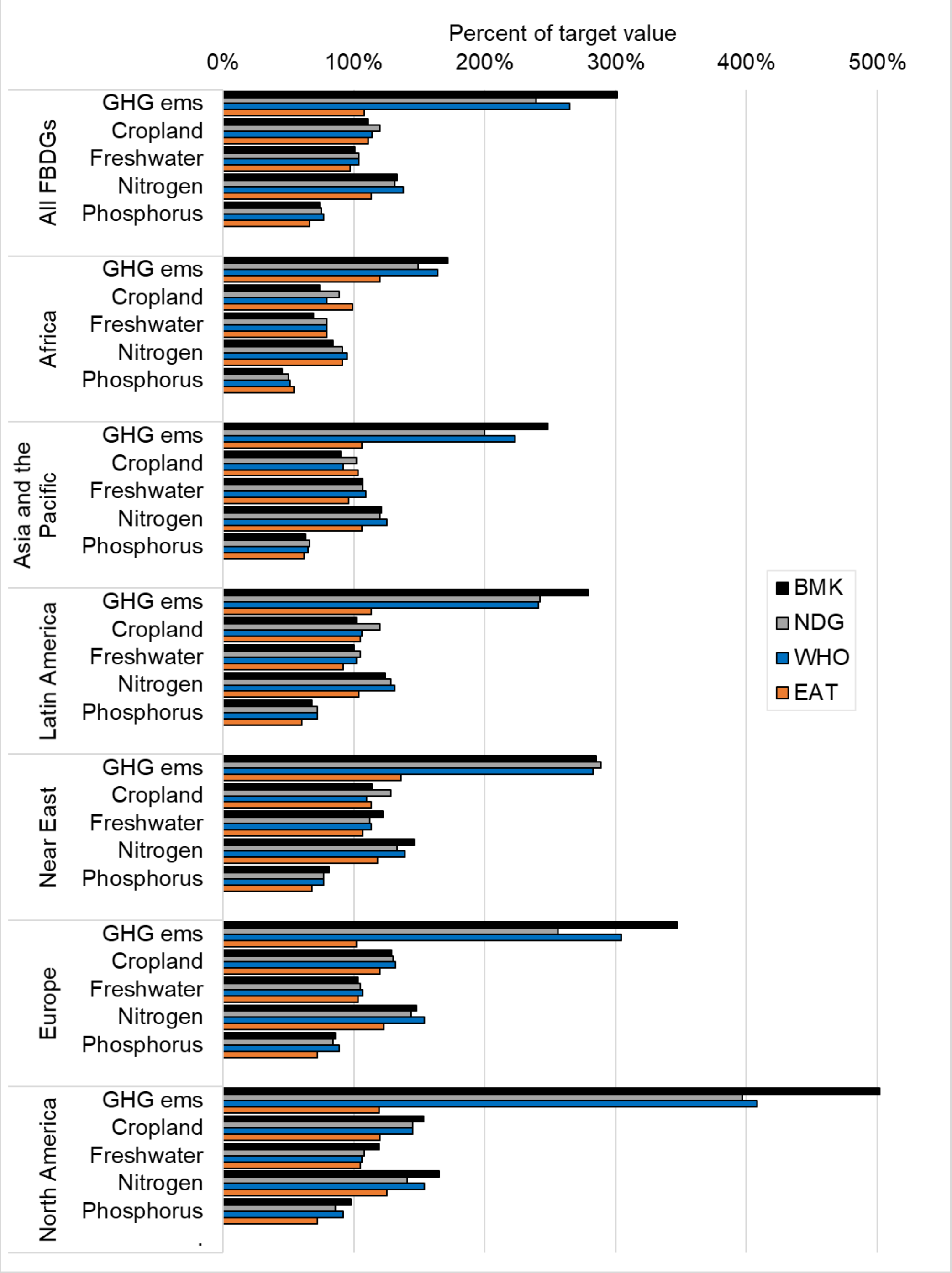

SI Figure 9. National FBDGs that when universally adopted fulfil the global health and environmental targets.

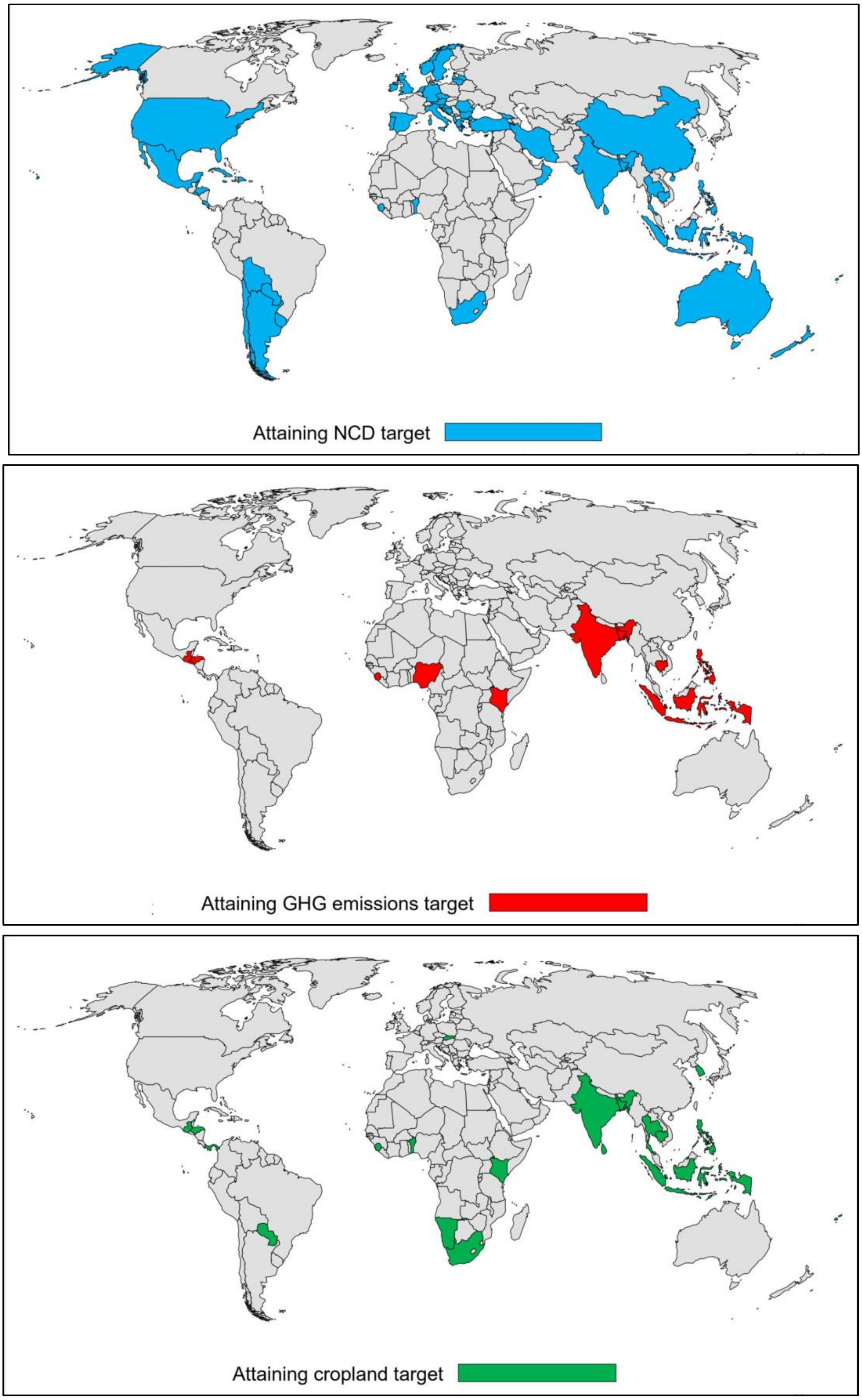

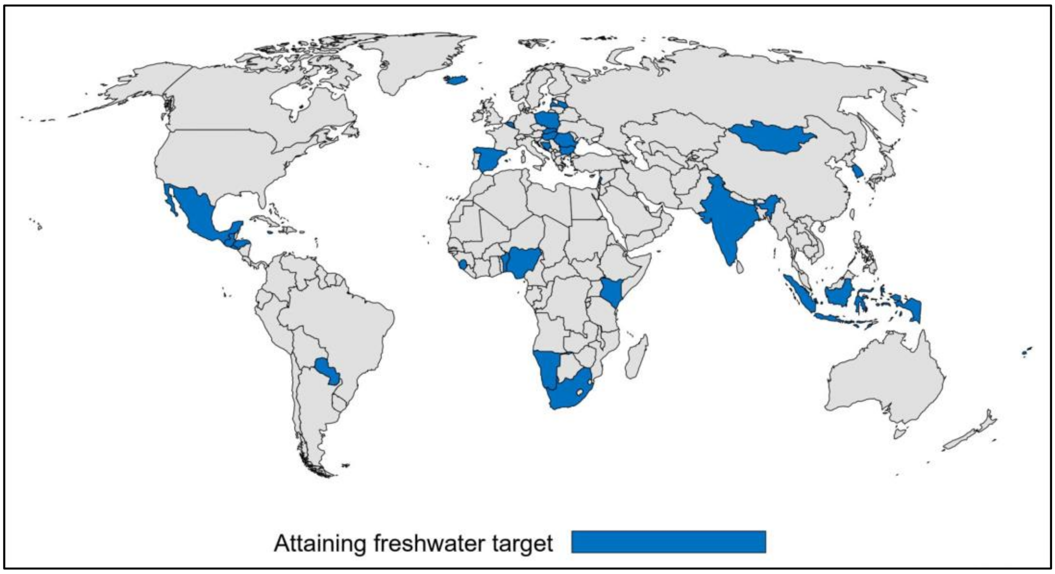
Attaining freshwater target

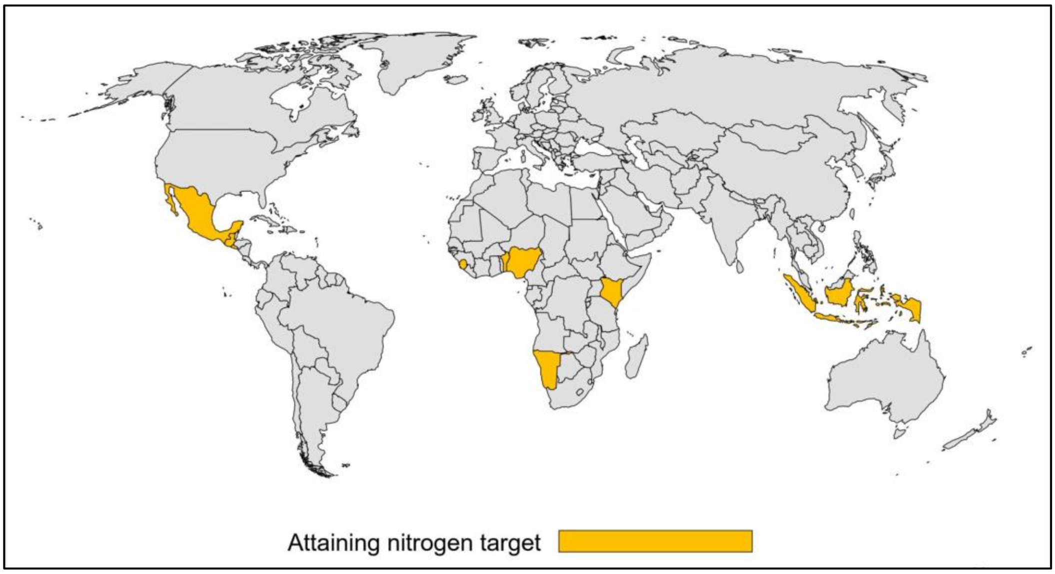
Attaining nitrogen target

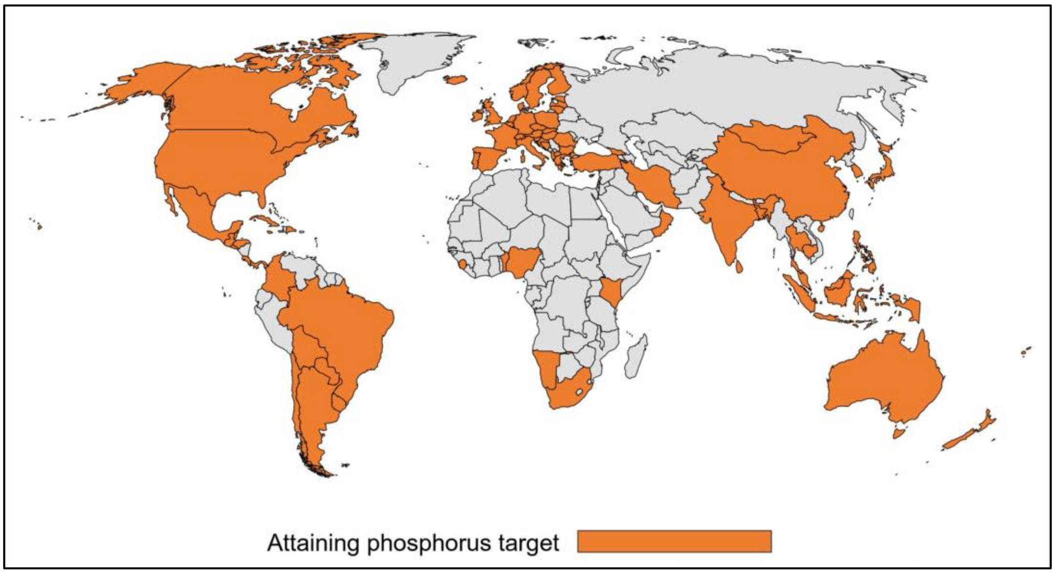
Attaining phosphorus target

SI.7 Uncertainty analysis

Our main representation of the FBDG guidelines used the mean of the range of recommended intake, and when food groups that are encouraged from a health perspective (fruits and vegetables, legumes, nuts and seeds, whole grains, and fish) were higher for baseline intake than recommended, then those were kept at that level and not reduced, and vice versa for the discouraged food groups (red meat, processed meat, and sugar). For analysing the uncertainty of quantitatively representing the FBDGs, we used the upper and lower values of the range of recommended intake. Simultaneous adoption of all low values and all high values was incompatible with attaining calorie balance in FBDGs that included a recommendation to balance energy intake. For that reasons, we used the high and low values of the recommendations to construct what from health and environmental perspectives can be considered low and high-impact representations that either emphasized the recommended (mostly plant-based) food groups over the discouraged and neutral (mostly animal-sourced) ones, and vice versa. In addition, we relaxed the non-adjustment rule for encouraged and discouraged foods used in the main representations to obtain rigorous ordering of consumption values and the largest possible range of representations.

SI Table 14. Consumption, health and environmental analyses of different representations of national FBDGs, including the main non-penalising representation (NDG, main), as well as low-impact (NDG, low), high-impact (NDG, high), and the mean of the non-adjusted recommendations (NDG, mean). BMK denotes baseline values.

Food group	BMK	NDG	NDG (non-adjusted)			WHO	WHO (non-adjusted)		
		(main)	mean	low	high	(main)	mean	low	high
Consumption (g/d)									
fruit&veg	437	586	515	609	457	508	400	480	400
> fruits	156	216	209	269	201	191	167	200	167
> vegetables	281	371	306	340	256	318	233	280	233
legumes	22	57	57	68	49	22	22	22	22
nuts&seeds	10	12	12	14	11	10	10	10	10
whole grains	43	97	96	138	86	149	147	308	145
milk	230	367	367	344	377	230	230	230	230
eggs	26	31	31	28	33	26	26	26	26
fish	27	38	36	47	28	27	27	27	27
sugar	46	31	43	34	44	37	50	30	50
meat	84	58	60	59	80	77	77	72	83
> red meat	44	27	29	27	36	43	44	44	44
> processed meat	13	7	7	7	13	6	6		11
> poultry	28	24	24	25	31	28	28	28	28
Reduction in premature mortality (%)									
mean risk values		14.7	14.0	17.5	11.9	14.8	13.1	15.0	12.6
low risk values		13.4	12.7	15.8	10.7	13.3	11.5	13.4	11.1
high risk values		15.9	15.3	19.2	13.1	16.3	14.6	16.7	14.1
Environmental resource demand									
GHG emissions (MtCO_2eq)	4,230	3,680	3,994	3,761	5,218	3,713	3,716	3,468	3,993
Cropland use (Mkm^2)	7,167	7,753	7,703	7,892	7,669	7,144	6,999	7,111	7,056
Freshwater use (km^3)	1,094	1,089	1,075	1,100	1,072	1,105	1,081	1,063	1,081
Nitrogen use (GgN)	65,006	64,846	63,032	64,862	62,650	65,038	61,169	62,413	61,559
Phosphorus use (GgP)	11,503	11,868	11,586	11,853	11,565	11,556	10,974	11,170	11,054
Global impacts (% of global target, average across countries with FBDGs)									
GHG emissions target	301%	239%	252%	239%	276%	265%	265%	244%	286%
Land-use target	111%	120%	119%	120%	121%	114%	113%	114%	114%
Water-use target	101%	104%	103%	105%	102%	104%	103%	104%	103%
Nitrogen target	133%	131%	131%	131%	133%	138%	136%	139%	137%
Phosphorus target	74%	75%	75%	75%	76%	77%	76%	78%	77%

SI Table 15. Consumption, health and environmental analyses of different representations of the EAT-Lancet recommendations, including flexitarian (FLX), pescatarian (PSC), vegetarian (VEG), and vegan (VGN) dietary patterns, as well as other global dietary recommendations (GDGs), including those by the World Health Organization (WHO) and the World Cancer Research Fund (WCRF). BMK denotes baseline values.

Food group	BMK	EAT-Lancet diets				GDGs	
		FLX	PSC	VEG	VGN	WHO	WCRF
Consumption (g/d)							
fruit&veg	437	597	669	745	829	508	508
> fruits	156	210	226	253	300	191	191
> vegetables	281	387	442	492	529	318	318
legumes	22	75	75	100	125	22	22
nuts&seeds	10	50	50	50	50	10	10
whole grains	43	198	204	186	198	149	153
milk	230	250	250	250		230	230
eggs	26	13	13	13		26	26
fish	27	35	57			27	27
sugar	46	25	25	25	25	37	37
meat	84	41				77	65
> red meat	44	12				43	33
> processed meat	13					6	4
> poultry	28	29				28	28
Reduction in premature mortality (%)							
mean risk values		19.7	20.5	21.1	22.3	14.8	15.2
low risk values		17.8	18.4	19.1	20.2	13.3	13.7
high risk values		21.6	22.6	23.0	24.5	16.3	16.7
Environmental resource demand							
GHG emissions ($MtCO_2eq$)	4,230	2,457	1,490	1,482	773	3,713	3,219
Cropland use (Mkm^2)	7,167	7,835	7,399	7,548	7,250	7,144	7,036
Freshwater use (km^3)	1,094	984	983	991	1,028	1,105	1,104
Nitrogen use (GgN)	65,006	55,385	53,079	51,466	49,730	65,038	64,284
Phosphorus use (GgP)	11,503	10,498	10,085	9,828	9,234	11,556	11,389
Global impacts (% of global target, sum across all countries)							
GHG emissions target	143%	99%	60%	60%	24%	129%	118%
Land-use target	79%	100%	94%	96%	91%	88%	87%
Water-use target	75%	82%	81%	83%	86%	87%	87%
Nitrogen target	104%	101%	96%	94%	88%	114%	113%
Phosphorus target	57%	59%	57%	56%	52%	61%	60%

SI Figure 10. Number of national FBDGs attaining zero to all six of the global health and environmental targets for the mean, high, and low values of the uncertainty range of the targets (SI Table 10).

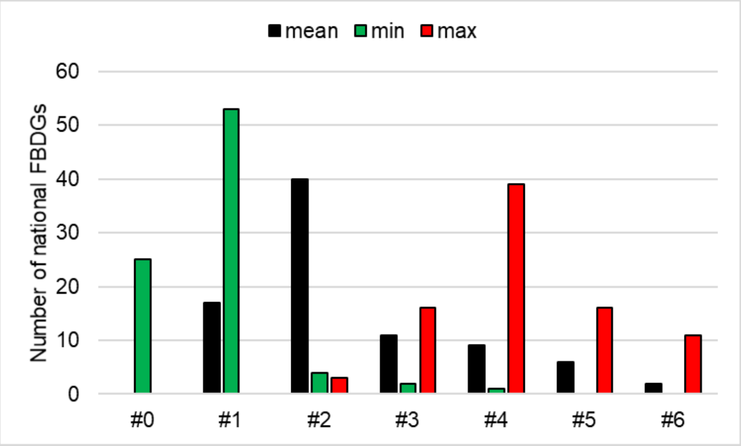

SI.8 Sensitivity analysis

SI Table 16. Percentage reductions in premature deaths for adoption of national FBDGs (NDG), and the WHO and EAT-Lancet recommendations, averaged over countries with national FBDGs for the main scenarios and a sensitivity analysis that included recommendations on fatty-acid intake coded in a binary fashion: if it was suggested to increase or prefer polyunsaturated fatty acids (PUFAs) and to decrease saturated fats, then any saturated fat intake above 10% was replaced by PUFAs, in line with WHO recommendations. For the health analysis, we used relative risk estimates for changes in PUFAs from Farvid and colleagues, and baseline consumption data from Micha and colleagues.[108,155]

Risk factors	Main results			With changes in fatty-acid composition		
	NDG	WHO	EAT	NDG	WHO	EAT
All risks	14.65	14.81	19.66	14.84	15.10	19.83
weight	7.23	7.71	7.71	7.23	7.71	7.71
diet	8.62	8.37	14.13	8.86	8.75	14.35
obese	3.19	3.49	3.49	3.19	3.49	3.49
overweight	1.80	1.94	1.94	1.80	1.94	1.94
underweight	2.24	2.29	2.29	2.24	2.29	2.29
whole grains	3.17	6.05	6.43	3.17	6.05	6.43
vegetables	1.92	0.96	2.50	1.92	0.96	2.50
fruits	1.66	1.00	1.75	1.66	1.00	1.75
nuts&seeds	0.21		1.91	0.21		1.91
legumes	0.85		1.57	0.85		1.57
processed meat	0.60	0.72	1.30	0.60	0.72	1.30
red meat	0.59	0.03	1.09	0.59	0.03	1.09
fish	0.49	0.00	0.51	0.49	0.00	0.51
PUFA				0.40	0.48	0.48

SI Table 17. Regions with national FBDGs

Region	Proportion of countries with FBDGs (%)	Proportion of countries without FBDGs (%)
Global	45	55
High-income countries	69	31
Upper middle-income countries	66	34
Lower middle-income countries	31	69
Low-income countries	12	88
Africa	13	87
Americas	71	29
Eastern Mediterranean	14	86
Europe	71	29
South-East Asia	45	55
Western Pacific	40	60

SI Table 18. Environmental and health impacts for adoption of the EAT-Lancet flexitarian diets in countries with national FBDGs versus global adoption

	Baseline diets		EAT-Lancet recommendations		
Parameter	in countries with FBDGs	globally	in countries with FBDGs	global impacts if adopted only in countries with national FBDGs	global adoption
Environmental resource demand					
GHG emissions ($MtCO_2eq$)	4,230	5,572	2,457	3,800	3,470
Cropland use (Mkm^2)	7,167	9,940	7,835	10,608	11,105
Freshwater use (km^3)	1,094	1,493	984	1,384	1,496
Nitrogen use (GgN)	65,006	76,075	55,385	66,454	66,393
Phosphorus use (GgP)	11,503	13,175	10,498	12,170	12,260
Reduction in premature mortality (thousands)					
Avoided premature deaths (mean)			2,834	2,834	3,803
Avoided premature deaths (low)			2,562	2,562	3,425
Avoided premature deaths (high)			3,106	3,106	4,182

SI.9 Supplementary economic analysis

In addition to reporting changes in mortality, we also estimated their economic value using estimates of the value of statistical life.[156] The value of statistical life (VSL) is a measure for the willingness to pay for a mortality risk reduction defined as the marginal rate of substitution between money and mortality risk in a defined time period [157]. The VSL does not represent the value of life itself, but rather the value of small risks to life which can be estimated either from market decisions that reveal the implicit values reflected in behaviour (revealed preference studies), or by using surveys which elicit respondents' willingness to pay for small reductions in mortality risks directly (stated preference studies).

We based our valuation on a comprehensive global meta-analysis of stated preference surveys of mortality risk valuation undertaken for the Organisation for Economic Co-operation and Development (OECD) [158]. Following OECD recommendations, we adopted a VSL base value for the EU-27 of USD 3.5 million (1.75-5.25 million) and used the benefit-transfer method to calculate VSLs in other regions [157]. In the benefit-transfer method, the VSL base value is adjusted by income (Y) subject to an elasticity of substitution (β):

$$VSL_r = VSL_{base}\left(\frac{Y_r}{Y_{base}}\right)^{\beta}$$

Following OECD recommendations, we used GDP per capita adjusted for purchasing power parity (PPP) as a proxy for income, and we adopted an elasticity of 0.8 for benefit transfers to high-income countries and an elasticity of 1.0 for benefit transfers to low and middle-income countries [157]. Baseline data on GDP per capita were sourced from the World Bank Development Indicator database. In line with World Bank methodology, we defined the income classification of countries depending on their GDP per capita (adjusted for purchasing power parity). SI Figure 11 provides an overview of the VSL estimates derived for this study.

SI Figure 12 shows the results of applying those to the changes in mortality for the different FBDG scenarios. The economic value of the reductions in mortality from adopting the national and global FBDGs amounted to USD 6-9.5 trillion globally, representing 10-16% of global GDP. In line with the health impacts, the economic value was greatest for adoption of the EAT-Lancet guidelines and lowest for the WHO ones. Across regions, the economic value ranged from 6-13% of GDP in Asia and the Pacific and Africa to 13-20% of GDP in Europe and North America, which reflects both the distribution of health benefits and the regional differences in the value of statistical life ascribed to those.

SI Figure 11. Value of statistical life (USD million) by region for the year 2010.

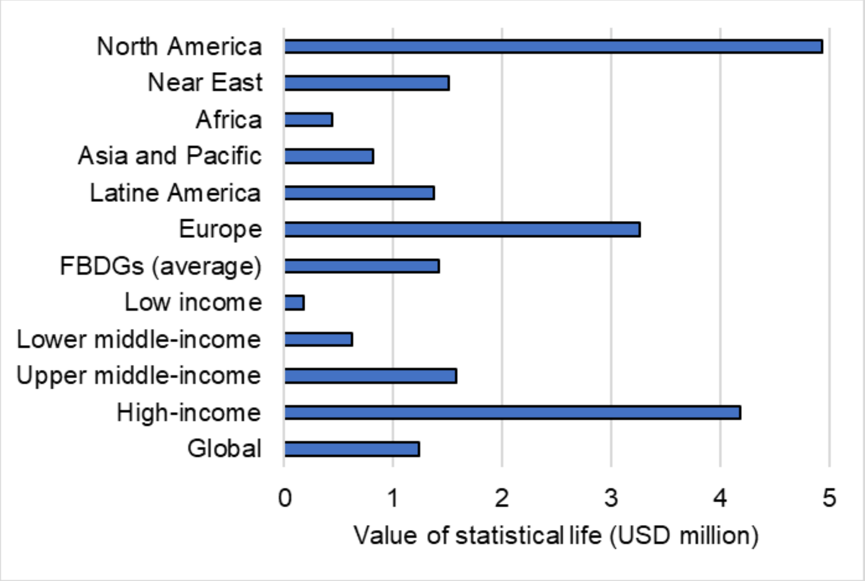

SI Figure 12. Economic value of adopting FBDGs by region. The FBDGs include national FBDGs (NDG), the EAT-Lancet recommendations on healthy and sustainable diets (EAT), and WHO recommendations (WHO). Total refers to the economic value of all countries FBDGs adopt those.

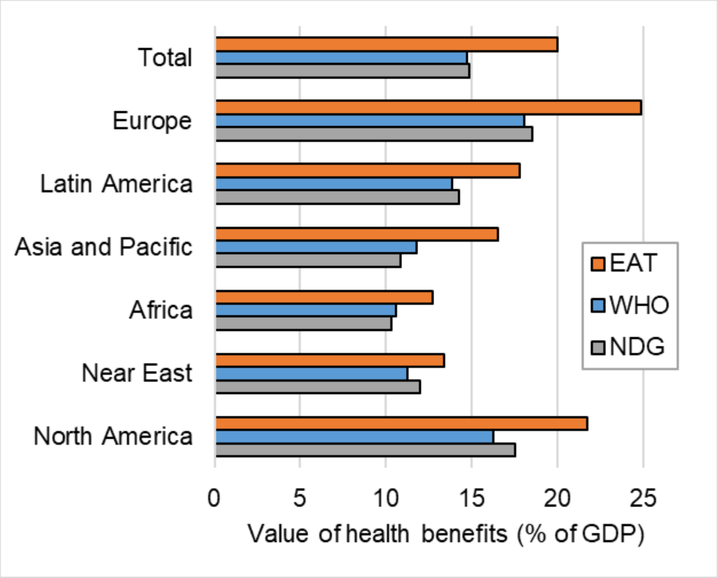

References

1. Springmann, M. *et al.* Health and nutritional aspects of sustainable diet strategies and their relationship to environmental impacts – a comparative global modelling analysis with country-level detail. *Lancet Planet. Heal.* **2**, e451–e461 (2018).
2. World Health Organization. *Human energy requirements: Report of a Joint FAO/WHO/UNU Expert Consultation, Rome, Italy, 17-24 October 2001*. (WHO, 2004).
3. Health, U. S. D. of & Services, H. *Dietary guidelines for Americans 2015-2020*. (Skyhorse Publishing Inc., 2017).
4. KC, S. & Lutz, W. The human core of the shared socioeconomic pathways: Population scenarios by age, sex and level of education for all countries to 2100. *Glob. Environ. Chang.* (2015). doi:10.1016/j.gloenvcha.2014.06.004
5. Wang, H. *et al.* Global, regional, and national life expectancy, all-cause mortality, and cause-specific mortality for 249 causes of death, 1980–2015: a systematic analysis for the Global Burden of Disease Study 2015. *Lancet* **388**, 1459–1544 (2016).
6. Gustavsson, J., Cederberg, C., Sonesson, U., Van Otterdijk, R. & Meybeck, A. *Global food losses and food waste: extent, causes and prevention*. (FAO Rome, 2011).
7. Food and Agriculture Organization of the United Nations. *Food balance sheets: a handbook*. (2001).
8. NCD Risk Factor Collaboration (NCD-RisC). Trends in adult body-mass index in 200 countries from 1975 to 2014: a pooled analysis of 1698 population-based measurement studies with 19·2 million participants. *Lancet* **387**, 1377–1396 (2016).
9. Micha, R. *et al.* Global, regional and national consumption of major food groups in 1990 and 2010: a systematic analysis including 266 country-specific nutrition surveys worldwide. *BMJ Open* **5**, e008705 (2015).
10. Murray, C. J. L., Ezzati, M., Lopez, A. D., Rodgers, A. & Vander Hoorn, S. Comparative quantification of health risks: conceptual framework and methodological issues. *Popul. Health Metr.* **1**, 1 (2003).
11. Lim, S. S. *et al.* A comparative risk assessment of burden of disease and injury attributable to 67 risk factors and risk factor clusters in 21 regions, 1990–2010: a systematic analysis for the Global Burden of Disease Study 2010. *Lancet* **380**, 2224–2260 (2012).
12. Forouzanfar, M. H. *et al.* Global, regional, and national comparative risk assessment of 79 behavioural, environmental and occupational, and metabolic risks or clusters of risks in 188 countries, 1990–2013: a systematic analysis for the Global Burden of Disease Study 2013. *Lancet* **386**, 2287–2323 (2015).
13. Murray, C. J. L. *et al.* GBD 2010: design, definitions, and metrics. *Lancet* **380**, 2063–2066 (2012).
14. Lozano, R. *et al.* Global and regional mortality from 235 causes of death for 20 age groups in 1990 and 2010: a systematic analysis for the Global Burden of Disease Study 2010. *Lancet* **380**, 2095–2128 (2012).
15. DeSA, U. N. World population prospects: the 2012 revision. *Popul. Div. Dep. Econ. Soc. Aff. United Nations Secr. New York* (2013).
16. Afshin, A., Micha, R., Khatibzadeh, S. & Mozaffarian, D. Consumption of nuts and legumes and risk of incident ischemic heart disease, stroke, and diabetes: a systematic review and meta-analysis. *Am. J. Clin. Nutr.* ajcn.076901 (2014). doi:10.3945/ajcn.113.076901
17. Aune, D. *et al.* Nut consumption and risk of cardiovascular disease, total cancer, all-cause and cause-specific mortality: a systematic review and dose-response meta-analysis of prospective studies. *BMC Med.* **14**, 207 (2016).
18. Aune, D. *et al.* Fruit and vegetable intake and the risk of cardiovascular disease, total cancer and all-cause mortality–a systematic review and dose-response meta-analysis of prospective studies. *Int. J. Epidemiol.* (2016).
19. Bechthold, A. *et al.* Food groups and risk of coronary heart disease, stroke and heart

failure: A systematic review and dose-response meta-analysis of prospective studies. *Crit. Rev. Food Sci. Nutr.* **59**, 1071–1090 (2019).
20. Schwingshackl, L. *et al.* Food groups and risk of type 2 diabetes mellitus: a systematic review and meta-analysis of prospective studies. *Eur. J. Epidemiol.* **32**, 363–375 (2017).
21. Schwingshackl, L. *et al.* Food groups and risk of colorectal cancer. *Int. J. Cancer* **142**, 1748–1758 (2018).
22. Zheng, J. *et al.* Fish consumption and CHD mortality: an updated meta-analysis of seventeen cohort studies. *Public Health Nutr.* **15**, 725–737 (2012).
23. Global BMI Mortality Collaboration, E. Di *et al.* Body-mass index and all-cause mortality: individual-participant-data meta-analysis of 239 prospective studies in four continents. *Lancet (London, England)* **388**, 776–86 (2016).
24. Aune, D. *et al.* Whole grain consumption and risk of cardiovascular disease, cancer, and all cause and cause specific mortality: systematic review and dose-response meta-analysis of prospective studies. *BMJ* **353**, i2716 (2016).
25. Singh, G. M. *et al.* The Age-Specific Quantitative Effects of Metabolic Risk Factors on Cardiovascular Diseases and Diabetes: A Pooled Analysis. *PLoS One* **8**, e65174 (2013).
26. Micha, R. *et al.* Etiologic effects and optimal intakes of foods and nutrients for risk of cardiovascular diseases and diabetes: Systematic reviews and meta-analyses from the Nutrition and Chronic Diseases Expert Group (NutriCoDE). *PLoS One* **12**, e0175149 (2017).
27. GBD 2017 Diet Collaborators, A. *et al.* Health effects of dietary risks in 195 countries, 1990-2017: a systematic analysis for the Global Burden of Disease Study 2017. *Lancet (London, England)* **0**, (2019).
28. Schwingshackl, L. *et al.* Perspective: NutriGrade: A Scoring System to Assess and Judge the Meta-Evidence of Randomized Controlled Trials and Cohort Studies in Nutrition Research. *Adv. Nutr. An Int. Rev. J.* **7**, 994–1004 (2016).
29. World Cancer Research Fund/American Institute for Cancer Research. Diet, Nutrition, Physical Activity and Cancer: A Global Perspective. Continuous Update Project Expert Report. (2018).
30. Aune, D., Norat, T., Romundstad, P. & Vatten, L. J. Dairy products and the risk of type 2 diabetes: a systematic review and dose-response meta-analysis of cohort studies. *Am. J. Clin. Nutr.* **98**, 1066–1083 (2013).
31. Aune, D. *et al.* Dairy products and colorectal cancer risk: a systematic review and meta-analysis of cohort studies. *Ann. Oncol. Off. J. Eur. Soc. Med. Oncol.* **23**, 37–45 (2012).
32. Micha, R., Wallace, S. K. & Mozaffarian, D. Red and processed meat consumption and risk of incident coronary heart disease, stroke, and diabetes mellitus: a systematic review and meta-analysis. *Circulation* **121**, 2271–2283 (2010).
33. Kaluza, J., Wolk, A. & Larsson, S. C. Red meat consumption and risk of stroke: a meta-analysis of prospective studies. *Stroke.* **43**, 2556–2560 (2012).
34. Yang, C. *et al.* Red Meat Consumption and the Risk of Stroke: A Dose-Response Meta-analysis of Prospective Cohort Studies. *J. Stroke Cerebrovasc. Dis. Off. J. Natl. Stroke Assoc.* **25**, 1177–1186 (2016).
35. Chen, G.-C., Lv, D.-B., Pang, Z. & Liu, Q.-F. Red and processed meat consumption and risk of stroke: a meta-analysis of prospective cohort studies. *Eur. J. Clin. Nutr.* **67**, 91–95 (2013).
36. Micha, R., Michas, G. & Mozaffarian, D. Unprocessed red and processed meats and risk of coronary artery disease and type 2 diabetes--an updated review of the evidence. *Curr. Atheroscler. Rep.* **14**, 515–524 (2012).
37. Feskens, E. J. M., Sluik, D. & van Woudenbergh, G. J. Meat consumption, diabetes, and its complications. *Curr. Diab. Rep.* **13**, 298–306 (2013).
38. Abete, I., Romaguera, D., Vieira, A. R., Lopez de Munain, A. & Norat, T. Association between total, processed, red and white meat consumption and all-cause, CVD and

IHD mortality: a meta-analysis of cohort studies. *Br. J. Nutr.* **112**, 762–775 (2014).
39. Wang, X. *et al.* Red and processed meat consumption and mortality: dose-response meta-analysis of prospective cohort studies. *Public Health Nutr.* **19**, 893–905 (2016).
40. Aune, D. *et al.* Red and processed meat intake and risk of colorectal adenomas: a systematic review and meta-analysis of epidemiological studies. *Cancer causes Control CCC* **24**, 611–627 (2013).
41. Bouvard, V. *et al.* Carcinogenicity of consumption of red and processed meat. *Lancet Oncol.* **16**, 1599–1600 (2015).
42. Larsson, S. C. & Wolk, A. Meat consumption and risk of colorectal cancer: a meta-analysis of prospective studies. *Int. J. Cancer* **119**, 2657–2664 (2006).
43. WCRF/AICR. *Food, Nutrition, Physical Activity, and the Prevention of Cancer: A Global Perspective*. (AICR, 2007).
44. Larsson, S. C. & Orsini, N. Red meat and processed meat consumption and all-cause mortality: a meta-analysis. *Am. J. Epidemiol.* kwt261 (2013).
45. Bechthold, A. *et al.* Food groups and risk of coronary heart disease, stroke and heart failure: A systematic review and dose-response meta-analysis of prospective studies. *Crit. Rev. Food Sci. Nutr.* **59**, 1071–1090 (2019).
46. Chan, D. S. M. *et al.* Red and processed meat and colorectal cancer incidence: meta-analysis of prospective studies. *PLoS One* **6**, e20456 (2011).
47. Schwingshackl, L. *et al.* Food groups and risk of all-cause mortality: A systematic review and meta-analysis of prospective studies. *American Journal of Clinical Nutrition* **105**, 1462–1473 (2017).
48. Wolk, A. Potential health hazards of eating red meat. *J. Intern. Med.* **281**, 106–122 (2017).
49. Bernstein, A. M. *et al.* Dietary protein sources and the risk of stroke in men and women. *Stroke* STROKEAHA-111 (2011).
50. Bernstein, A. M. *et al.* Major dietary protein sources and risk of coronary heart disease in women. *Circulation* **122**, 876–883 (2010).
51. Pan, A. *et al.* Red meat consumption and mortality: results from 2 prospective cohort studies. *Arch. Intern. Med.* **172**, 555–563 (2012).
52. Pan, A. *et al.* Red meat consumption and risk of type 2 diabetes: 3 cohorts of US adults and an updated meta-analysis. *Am. J. Clin. Nutr.* **94**, 1088–1096 (2011).
53. Larsson, S. C., Crippa, A., Orsini, N., Wolk, A. & Michaëlsson, K. Milk Consumption and Mortality from All Causes, Cardiovascular Disease, and Cancer: A Systematic Review and Meta-Analysis. *Nutrients* **7**, 7749–7763 (2015).
54. Mullie, P., Pizot, C. & Autier, P. Daily milk consumption and all-cause mortality, coronary heart disease and stroke: a systematic review and meta-analysis of observational cohort studies. *BMC Public Health* **16**, 1236 (2016).
55. Soedamah-Muthu, S. S. *et al.* Milk and dairy consumption and incidence of cardiovascular diseases and all-cause mortality: dose-response meta-analysis of prospective cohort studies. *Am. J. Clin. Nutr.* **93**, 158–171 (2011).
56. Aune, D. *et al.* Dairy products, calcium, and prostate cancer risk: a systematic review and meta-analysis of cohort studies. *Am. J. Clin. Nutr.* **101**, 87–117 (2015).
57. Lu, W. *et al.* Dairy products intake and cancer mortality risk: a meta-analysis of 11 population-based cohort studies. *Nutr. J.* **15**, 91 (2016).
58. Alexander, D. D. *et al.* Dairy consumption and CVD: a systematic review and meta-analysis. *Br. J. Nutr.* **115**, 737–750 (2016).
59. de Goede, J., Soedamah-Muthu, S. S., Pan, A., Gijsbers, L. & Geleijnse, J. M. Dairy Consumption and Risk of Stroke: A Systematic Review and Updated Dose-Response Meta-Analysis of Prospective Cohort Studies. *J. Am. Heart Assoc.* **5**, (2016).
60. Gijsbers, L. *et al.* Consumption of dairy foods and diabetes incidence: a dose-response meta-analysis of observational studies. *Am. J. Clin. Nutr.* **103**, 1111–1124 (2016).
61. Ludwig, D. S. & Willett, W. C. Three daily servings of reduced-fat milk: an evidence-based recommendation? *JAMA Pediatr.* **167**, 788–789 (2013).

62. Bolland, M. J. *et al.* Calcium intake and risk of fracture: systematic review. *BMJ* **351**, h4580 (2015).
63. Bischoff-Ferrari, H. A. *et al.* Milk intake and risk of hip fracture in men and women: a meta-analysis of prospective cohort studies. *J. Bone Miner. Res. Off. J. Am. Soc. Bone Miner. Res.* **26**, 833–839 (2011).
64. Tai, V., Leung, W., Grey, A., Reid, I. R. & Bolland, M. J. Calcium intake and bone mineral density: systematic review and meta-analysis. *BMJ* **351**, h4183 (2015).
65. Kretchmer, N. Lactose and lactase--a historical perspective. *Gastroenterology* **61**, 805–813 (1971).
66. Lomer, M. C. E., Parkes, G. C. & Sanderson, J. D. Review article: lactose intolerance in clinical practice--myths and realities. *Aliment. Pharmacol. Ther.* **27**, 93–103 (2008).
67. Scrimshaw, N. S. & Murray, E. B. The acceptability of milk and milk products in populations with a high prevalence of lactose intolerance. *Am. J. Clin. Nutr.* **48**, 1079–1159 (1988).
68. Deng, Y., Misselwitz, B., Dai, N. & Fox, M. Lactose Intolerance in Adults: Biological Mechanism and Dietary Management. *Nutrients* **7**, 8020–8035 (2015).
69. He, K. *et al.* Accumulated evidence on fish consumption and coronary heart disease mortality: a meta-analysis of cohort studies. *Circulation* **109**, 2705–2711 (2004).
70. Larsson, S. C. & Orsini, N. Fish consumption and the risk of stroke: a dose-response meta-analysis. *Stroke* **42**, 3621–3623 (2011).
71. Xun, P. *et al.* Fish consumption and risk of stroke and its subtypes: accumulative evidence from a meta-analysis of prospective cohort studies. *Eur. J. Clin. Nutr.* **66**, 1199–1207 (2012).
72. Zhao, L.-G. *et al.* Fish consumption and all-cause mortality: a meta-analysis of cohort studies. *Eur. J. Clin. Nutr.* **70**, 155–161 (2016).
73. Wallin, A. *et al.* Fish consumption, dietary long-chain n-3 fatty acids, and risk of type 2 diabetes: systematic review and meta-analysis of prospective studies. *Diabetes Care* **35**, 918–929 (2012).
74. Zhang, M., Picard-Deland, E. & Marette, A. Fish and marine omega-3 polyunsatured Fatty Acid consumption and incidence of type 2 diabetes: a systematic review and meta-analysis. *Int. J. Endocrinol.* **2013**, 501015 (2013).
75. Del Gobbo, L. C. *et al.* ω-3 Polyunsaturated Fatty Acid Biomarkers and Coronary Heart Disease: Pooling Project of 19 Cohort Studies. *JAMA Intern. Med.* **176**, 1155–1166 (2016).
76. Afshin, A., Micha, R., Khatibzadeh, S. & Mozaffarian, D. Consumption of nuts and legumes and risk of incident ischemic heart disease, stroke, and diabetes: a systematic review and meta-analysis. *Am. J. Clin. Nutr.* **100**, 278–288 (2014).
77. Luo, C. *et al.* Nut consumption and risk of type 2 diabetes, cardiovascular disease, and all-cause mortality: a systematic review and meta-analysis. *Am. J. Clin. Nutr.* **100**, 256–269 (2014).
78. Mayhew, A. J., de Souza, R. J., Meyre, D., Anand, S. S. & Mente, A. A systematic review and meta-analysis of nut consumption and incident risk of CVD and all-cause mortality. *Br. J. Nutr.* **115**, 212–225 (2016).
79. Grosso, G. *et al.* Nut consumption on all-cause, cardiovascular, and cancer mortality risk: a systematic review and meta-analysis of epidemiologic studies. *Am. J. Clin. Nutr.* **101**, 783–793 (2015).
80. Shi, Z. Q., Tang, J. J., Wu, H., Xie, C. Y. & He, Z. Z. Consumption of nuts and legumes and risk of stroke: a meta-analysis of prospective cohort studies. *Nutr. Metab. Cardiovasc. Dis. NMCD* **24**, 1262–1271 (2014).
81. Zhou, D. *et al.* Nut consumption in relation to cardiovascular disease risk and type 2 diabetes: a systematic review and meta-analysis of prospective studies. *Am. J. Clin. Nutr.* **100**, 270–277 (2014).
82. Zhu, B., Sun, Y., Qi, L., Zhong, R. & Miao, X. Dietary legume consumption reduces risk of colorectal cancer: evidence from a meta-analysis of cohort studies. *Sci. Rep.* **5**, 8797 (2015).

83. Dauchet, L., Amouyel, P., Hercberg, S. & Dallongeville, J. Fruit and vegetable consumption and risk of coronary heart disease: a meta-analysis of cohort studies. *J. Nutr.* **136**, 2588–2593 (2006).
84. Gan, Y. *et al.* Consumption of fruit and vegetable and risk of coronary heart disease: A meta-analysis of prospective cohort studies. *Int. J. Cardiol.* **183**, 129–137 (2015).
85. He, F. J., Nowson, C. A., Lucas, M. & MacGregor, G. A. Increased consumption of fruit and vegetables is related to a reduced risk of coronary heart disease: meta-analysis of cohort studies. *J. Hum. Hypertens.* **21**, 717–728 (2007).
86. Dauchet, L., Amouyel, P. & Dallongeville, J. Fruit and vegetable consumption and risk of stroke: a meta-analysis of cohort studies. *Neurology* **65**, 1193–1197 (2005).
87. Hu, D., Huang, J., Wang, Y., Zhang, D. & Qu, Y. Fruits and Vegetables Consumption and Risk of Stroke. *Stroke* **45**, 1613–1619 (2014).
88. Li, M., Fan, Y., Zhang, X., Hou, W. & Tang, Z. Fruit and vegetable intake and risk of type 2 diabetes mellitus: meta-analysis of prospective cohort studies. *BMJ Open* **4**, e005497 (2014).
89. Wu, Y., Zhang, D., Jiang, X. & Jiang, W. Fruit and vegetable consumption and risk of type 2 diabetes mellitus: a dose-response meta-analysis of prospective cohort studies. *Nutr. Metab. Cardiovasc. Dis. NMCD* **25**, 140–147 (2015).
90. Wang, X. *et al.* Fruit and vegetable consumption and mortality from all causes, cardiovascular disease, and cancer: systematic review and dose-response meta-analysis of prospective cohort studies. *BMJ* **349**, g4490 (2014).
91. Wang, X. *et al.* Fruit and vegetable consumption and mortality from all causes, cardiovascular disease, and cancer: systematic review and dose-response meta-analysis of prospective cohort studies. *BMJ* **349**, g4490–g4490 (2014).
92. Vieira, A. R. *et al.* Fruits, vegetables and lung cancer risk: a systematic review and meta-analysis. *Ann. Oncol.* mdv381 (2015). doi:10.1093/annonc/mdv381
93. Bjelakovic, G., Nikolova, D., Gluud, L. L., Simonetti, R. G. & Gluud, C. Antioxidant supplements for prevention of mortality in healthy participants and patients with various diseases. *Cochrane Database Syst. Rev.* CD007176 (2012). doi:10.1002/14651858.CD007176.pub2
94. Schwingshackl, L. *et al.* Dietary Supplements and Risk of Cause-Specific Death, Cardiovascular Disease, and Cancer: A Systematic Review and Meta-Analysis of Primary Prevention Trials. *Adv. Nutr.* **8**, 27–39 (2017).
95. Liu, R. H. Health-Promoting Components of Fruits and Vegetables in the Diet. *Adv. Nutr. An Int. Rev. J.* **4**, 384S-392S (2013).
96. Liu, R. H. Dietary Bioactive Compounds and Their Health Implications. *J. Food Sci.* **78**, A18–A25 (2013).
97. Borgi, L., Rimm, E. B., Willett, W. C. & Forman, J. P. Potato intake and incidence of hypertension: results from three prospective US cohort studies. *BMJ* **353**, i2351 (2016).
98. King, J. C. & Slavin, J. L. White potatoes, human health, and dietary guidance. *Adv. Nutr.* **4**, 393S-401S (2013).
99. Muraki, I. *et al.* Potato Consumption and Risk of Type 2 Diabetes: Results From Three Prospective Cohort Studies. *Diabetes Care* **39**, 376–384 (2016).
100. Aune, D. *et al.* Whole grain consumption and risk of cardiovascular disease, cancer, and all cause and cause specific mortality: systematic review and dose-response meta-analysis of prospective studies. *BMJ* **353**, i2716 (2016).
101. Zong, G., Gao, A., Hu, F. B. & Sun, Q. Whole Grain Intake and Mortality From All Causes, Cardiovascular Disease, and Cancer: A Meta-Analysis of Prospective Cohort Studies. *Circulation* **133**, 2370–2380 (2016).
102. Ye, E. Q., Chacko, S. A., Chou, E. L., Kugizaki, M. & Liu, S. Greater whole-grain intake is associated with lower risk of type 2 diabetes, cardiovascular disease, and weight gain. *J. Nutr.* **142**, 1304–1313 (2012).
103. Aune, D., Norat, T., Romundstad, P. & Vatten, L. J. Whole grain and refined grain consumption and the risk of type 2 diabetes: a systematic review and dose–response

meta-analysis of cohort studies. *Eur. J. Epidemiol.* **28**, 845–858 (2013).
104. Chen, J. *et al.* Meta-Analysis of the Association Between Whole and Refined Grain Consumption and Stroke Risk Based on Prospective Cohort Studies. *Asia-Pacific J. Public Heal.* **28**, 563–575 (2016).
105. Hu, E. A., Pan, A., Malik, V. & Sun, Q. White rice consumption and risk of type 2 diabetes: meta-analysis and systematic review. *BMJ* **344**, e1454 (2012).
106. de Souza, R. J. *et al.* Intake of saturated and trans unsaturated fatty acids and risk of all cause mortality, cardiovascular disease, and type 2 diabetes: systematic review and meta-analysis of observational studies. *BMJ* **351**, h3978 (2015).
107. Mozaffarian, D. & Clarke, R. Quantitative effects on cardiovascular risk factors and coronary heart disease risk of replacing partially hydrogenated vegetable oils with other fats and oils. *Eur. J. Clin. Nutr.* **63 Suppl 2**, S22-33 (2009).
108. Farvid, M. S. *et al.* Dietary linoleic acid and risk of coronary heart disease: a systematic review and meta-analysis of prospective cohort studies. *Circulation* **130**, 1568–1578 (2014).
109. Jakobsen, M. U. *et al.* Major types of dietary fat and risk of coronary heart disease: a pooled analysis of 11 cohort studies. *Am. J. Clin. Nutr.* **89**, 1425–1432 (2009).
110. Mozaffarian, D., Micha, R. & Wallace, S. Effects on coronary heart disease of increasing polyunsaturated fat in place of saturated fat: a systematic review and meta-analysis of randomized controlled trials. *PLoS Med.* **7**, e1000252 (2010).
111. Hooper, L., Martin, N., Abdelhamid, A. & Davey Smith, G. Reduction in saturated fat intake for cardiovascular disease. *Cochrane Database Syst. Rev.* CD011737 (2015). doi:10.1002/14651858.CD011737
112. Mensink, R. P., Zock, P. L., Kester, A. D. M. & Katan, M. B. Effects of dietary fatty acids and carbohydrates on the ratio of serum total to HDL cholesterol and on serum lipids and apolipoproteins: a meta-analysis of 60 controlled trials. *Am. J. Clin. Nutr.* **77**, 1146–1155 (2003).
113. Micha, R. & Mozaffarian, D. Saturated fat and cardiometabolic risk factors, coronary heart disease, stroke, and diabetes: a fresh look at the evidence. *Lipids* **45**, 893–905 (2010).
114. Chen, M. *et al.* Dairy fat and risk of cardiovascular disease in 3 cohorts of US adults. *Am. J. Clin. Nutr.* **104**, 1209–1217 (2016).
115. Malik, V. S., Pan, A., Willett, W. C. & Hu, F. B. Sugar-sweetened beverages and weight gain in children and adults: a systematic review and meta-analysis. *Am. J. Clin. Nutr.* **98**, 1084–1102 (2013).
116. Te Morenga, L., Mallard, S. & Mann, J. Dietary sugars and body weight: systematic review and meta-analyses of randomised controlled trials and cohort studies. *BMJ* **346**, e7492 (2012).
117. Malik, V. S. *et al.* Sugar-sweetened beverages and risk of metabolic syndrome and type 2 diabetes: a meta-analysis. *Diabetes Care* **33**, 2477–2483 (2010).
118. Te Morenga, L. A., Howatson, A. J., Jones, R. M. & Mann, J. Dietary sugars and cardiometabolic risk: systematic review and meta-analyses of randomized controlled trials of the effects on blood pressure and lipids. *Am. J. Clin. Nutr.* **100**, 65–79 (2014).
119. Imamura, F. *et al.* Consumption of sugar sweetened beverages, artificially sweetened beverages, and fruit juice and incidence of type 2 diabetes: systematic review, meta-analysis, and estimation of population attributable fraction. *BMJ* **351**, h3576 (2015).
120. Malik, V. S. & Hu, F. B. Fructose and Cardiometabolic Health: What the Evidence From Sugar-Sweetened Beverages Tells Us. *J. Am. Coll. Cardiol.* **66**, 1615–1624 (2015).
121. WC, W., JE, M., MJ, S. & al, et. Weight, weight change, and coronary heart disease in women: Risk within the ‘normal’ weight range. *JAMA* **273**, 461–465 (1995).
122. Collaboration, A. P. C. S. Body mass index and cardiovascular disease in the Asia-Pacific Region: an overview of 33 cohorts involving 310 000 participants. *Int. J. Epidemiol.* **33**, 751–758 (2004).
123. Song, Y.-M., Sung, J., Smith, G. D. & Ebrahim, S. Body Mass Index and Ischemic and

Hemorrhagic Stroke A Prospective Study in Korean Men. *Stroke* **35**, 831–836 (2004).
124. KM, R., CH, H., WC, W. & al, et. A prospective study of body mass index, weight change, and risk of stroke in women. *JAMA* **277**, 1539–1545 (1997).
125. Calle, E. E., Rodriguez, C., Walker-Thurmond, K. & Thun, M. J. Overweight, obesity, and mortality from cancer in a prospectively studied cohort of U.S. adults. *N. Engl. J. Med.* **348**, 1625–1638 (2003).
126. Reeves, G. K. *et al.* Cancer incidence and mortality in relation to body mass index in the Million Women Study: cohort study. *BMJ* **335**, 1134 (2007).
127. Parr, C. L. *et al.* Body-mass index and cancer mortality in the Asia-Pacific Cohort Studies Collaboration: pooled analyses of 424,519 participants. *Lancet. Oncol.* **11**, 741–752 (2010).
128. Calle, E. E. & Kaaks, R. Overweight, obesity and cancer: epidemiological evidence and proposed mechanisms. *Nat. Rev. Cancer* **4**, 579–591 (2004).
129. Willett, W. C., Dietz, W. H. & Colditz, G. A. Guidelines for healthy weight. *N. Engl. J. Med.* **341**, 427–434 (1999).
130. Collaboration, P. S. *et al.* Body-mass index and cause-specific mortality in 900 000 adults: collaborative analyses of 57 prospective studies. *Lancet* **373**, 1083–1096 (2009).
131. Chiolero, A. & Kaufman, J. S. Metabolic mediators of body-mass index and cardiovascular risk. *Lancet* **383**, 2042 (2014).
132. Yusuf, S. *et al.* Effect of potentially modifiable risk factors associated with myocardial infarction in 52 countries (the INTERHEART study): case-control study. *Lancet* **364**, 937–952 (2004).
133. Khaw, K.-T. *et al.* Combined Impact of Health Behaviours and Mortality in Men and Women: The EPIC-Norfolk Prospective Population Study. *PLoS Med* **5**, e12 (2008).
134. Dam, R. M. van, Li, T., Spiegelman, D., Franco, O. H. & Hu, F. B. Combined Impact of Lifestyle Factors on Mortality: Prospective Cohort Study in US Women. *BMJ Br. Med. J.* **337**, 742–745 (2008).
135. Huxley, R. R. *et al.* The impact of dietary and lifestyle risk factors on risk of colorectal cancer: a quantitative overview of the epidemiological evidence. *Int. J. Cancer* **125**, 171–180 (2009).
136. Nechuta, S. J. *et al.* Combined impact of lifestyle-related factors on total and cause-specific mortality among Chinese women: prospective cohort study. *PLoS Med.* **7**, (2010).
137. Berrington de Gonzalez, A. *et al.* Body-Mass Index and Mortality among 1.46 Million White Adults. *N. Engl. J. Med.* **363**, 2211–2219 (2010).
138. Springmann, M. *et al.* Options for keeping the food system within environmental limits. *Nature* **562**, 519–525 (2018).
139. Robinson, S. *et al.* The International Model for Policy Analysis of Agricultural Commodities and Trade (IMPACT) -- Model description for version 3. (2015).
140. Tubiello, F. N. *et al. Agriculture, Forestry and Other Land Use Emissions by Sources and Removals by Sinks: 1990 - 2011 Analysis*. *ESS Working Paper No. 2, Mar 2014* (FAO Statistical Division, 2014).
141. Carlson, K. M. *et al.* Greenhouse gas emissions intensity of global croplands. *Nat. Clim. Chang.* **7**, 63–68 (2017).
142. Heffer, P. Assessment of Fertilizer Use by Crop at the Global Level 2010–2010/11. (2013).
143. Alexandratos, N. & Bruinsma, J. *World agriculture towards 2030/2050: the 2012 revision*. *ESA Working Paper No. 12-03* (FAO, 2012).
144. Tubiello, F. N. *et al.* The FAOSTAT database of greenhouse gas emissions from agriculture. *Environ. Res. Lett.* **8**, 15009 (2013).
145. Troell, M. *et al.* Does aquaculture add resilience to the global food system? *Proc. Natl. Acad. Sci.* **111**, 13257–13263 (2014).
146. Chan, C. Y. *et al. Fish to 2050 in the ASEAN Region*. (WorldFish Center and Intl Food Policy Res Inst, 2017).

147. Rosegrant, M. W. *et al. Quantitative foresight modeling to inform the CGIAR research portfolio*. (Intl Food Policy Res Inst, 2017).
148. Beach, R. H. *et al.* Global mitigation potential and costs of reducing agricultural non-CO2 greenhouse gas emissions through 2030. *J. Integr. Environ. Sci.* **12**, 87–105 (2015).
149. Group, I. W. *Technical update on the social cost of carbon for regulatory impact analysis-under executive order 12866*. (United States Government, 2013).
150. Mueller, N. D. *et al.* Closing yield gaps through nutrient and water management. *Nature* **490**, 254 (2012).
151. Sutton, M. A. *et al.* Our Nutrient World: The challenge to produce more food and energy with less pollution. Global Overview of Nutrient Management. Centre for Ecology and Hydrology, Edinburgh on behalf of the Global Partnership on Nutrient Management and the International Nit. *Edinburgh behalf Glob. Partnersh. Nutr. Manag. Int. Nitrogen Initiat.* (2013).
152. Cordell, D. & White, S. Life's bottleneck: sustaining the world's phosphorus for a food secure future. *Annu. Rev. Environ. Resour.* **39**, 161–188 (2014).
153. Wollenberg, E. *et al.* Reducing emissions from agriculture to meet the 2°C target. *Glob. Chang. Biol.* (2016). doi:10.1111/gcb.13340
154. GBD 2017 Risk Factor Collaborators *et al.* Global, regional, and national comparative risk assessment of 84 behavioural, environmental and occupational, and metabolic risks or clusters of risks for 195 countries and territories, 1990-2017: a systematic analysis for the Global Burden of Disease Stu. *Lancet* **392**, 1923–1994 (2018).
155. Micha, R. *et al.* Global, regional, and national consumption levels of dietary fats and oils in 1990 and 2010: a systematic analysis including 266 country-specific nutrition surveys. *BMJ* **348**, g2272–g2272 (2014).
156. Springmann, M., Godfray, H. C. J., Rayner, M. & Scarborough, P. Analysis and valuation of the health and climate change cobenefits of dietary change. *Proc. Natl. Acad. Sci.* **113**, 4146–4151 (2016).
157. OECD. *Mortality Risk Valuation in Environment, Health and Transport Policies*. (OECD, 2012).
158. Lindhjem, H., Navrud, S., Braathen, N. A. & Biausque, V. Valuing Mortality Risk Reductions from Environmental, Transport, and Health Policies: A Global Meta-Analysis of Stated Preference Studies. *Risk Anal.* **31**, 1381–1407 (2011).
